# Inhibition of mitochondrial respiration under hypoxia and increased antioxidant activity after reoxygenation of *Tribolium castaneum*

**DOI:** 10.1371/journal.pone.0199056

**Published:** 2018-06-14

**Authors:** Lei Wang, Sufen Cui, Zhicheng Liu, Yong Ping, Jiangping Qiu, Xueqing Geng

**Affiliations:** 1 School of Agriculture and Biology, Shanghai Jiao Tong University, Shanghai, PR China; 2 Bio-X institutes, Shanghai Jiao Tong University, Shanghai, PR China; CINVESTAV-IPN, MEXICO

## Abstract

Regulating the air in low-oxygen environments protects hermetically stored grains from storage pests damage. However, pests that can tolerate hypoxic stress pose a huge challenge in terms of grain storage. We used various biological approaches to determine the fundamental mechanisms of *Tribolium castaneum* to cope with hypoxia. Our results indicated that limiting the available oxygen to *T*. *castaneum* increased glycolysis and inhibited the Krebs cycle, and that accumulated pyruvic acid was preferentially converted to lactic acid via anaerobic metabolism. Mitochondrial aerobic respiration was markedly suppressed for beetles under hypoxia, which also might have led to mitochondrial autophagy. The enzymatic activity of citrate synthase decreased in insects under hypoxia but recovered within 12 h, which suggested that the beetles recovered from the hypoxia. Moreover, hypoxia-reperfusion resulted in severe oxidative damage to insects, and antioxidant levels increased to defend against the high level of reactive oxygen species. In conclusion, our findings show that mitochondria were the main target in *T*. *castaneum* in response to low oxygen. The beetles under hypoxia inhibited mitochondrial respiration and increased antioxidant activity after reoxygenation. Our research advances the field of pest control and makes it possible to develop more efficient strategies for hermetic storage.

## Introduction

Oxygen is essential for aerobic life to maintain normal energy production and homeostasis. Hypoxia, defined as a pathological condition in which the body or a part of the body is deprived of an adequate supply of oxygen, can have severe consequences for organisms [1]. It damages lipids, inhibits protein synthesis [2], and increases oxidative damage caused by reactive oxygen species (ROS) [3].

In recent years, studies on hypoxia/anoxia tolerance in insects have made great progress [4–9]. For example, *Manduca sexta* survives more than 24 h of anoxia, whereas *Locusta migratoria* recovers from 4 h of pure nitrogen anoxia [10]. Severe hypoxia also results in a decrease in ATP and production of lactate in larvae of the confamilial tiger beetle (Coleoptera: Cicindelidae) [11, 12]. The alder leaf beetle (*Agelastica alni* L.) survives 10 h of anoxia and recovers within 90 min [13]. The molecular mechanisms of adaptation and/or resistance to hypoxia have also been explored in *Drosophila melanogaster* [14, 15]. Fruit flies respond to hypoxia by limiting their energy demands, maintaining a balance between oxygen concentration and energy consumption. Limited ATP inhibits macromolecular synthesis, resulting in developmental delays and reductions in body size and weight [16, 17]. Many genes involved in cellular respiration and metabolism are downregulated during hypoxia of a long duration [18]. It is interesting that these genes principally encode lipid β-oxidation enzymes, respiratory chain complexes, and enzymes involved in the tricarboxylic acid (TCA) cycle [14]. Mitochondrial genes and translational regulators involved in hypoxia have been identified using an unbiased genome-wide RNA interference (RNAi) screen [19]. Specifically, hypoxia-selected flies display rearrangements in the mitochondrial ultrastructure. A few regions devoid of cristae are observed in the hypoxic mitochondria. These mitochondria have small and fractured cristae, and a dense core appears in the degenerated cristae. These results suggest that hypoxia can result in severe damage to the mitochondria of insects.

The hypoxia-inducible transcription factor (HIF), a member of the basic helix-loop-helix family of proteins that is a heterodimeric protein composed of alpha and beta subunits, is a critical regulator of cellular and systemic responses to hypoxia. HIF is induced when cells or tissues are exposed to low oxygen levels and regulates downstream genes to adapt or resist environmental hypoxia [20, 21]. Research on various species has illustrated that HIF is a ubiquitous and global regulator in response to hypoxia. The HIF pathway transduces decreased oxygen into changes in genetic activity and proteic functions in a conserved manner, from insects to nematodes to mammals [22]. However, whereas much information is available on the genetics of hypoxia in *D*. *melanogaster* and *C*. *elegans*, the molecular mechanisms of resistance to hypoxia in storage pests remain largely unknown.

The red flour beetle, *Tribolium castaneum* (Coleoptera: Tenebrionidae), is a major global pest of cereal products and stored grains [23]. Hermetic storage is widely used to protect grains from storage pests damage. Dry food crops are stored in air-tight facilities under conditions of hypoxia and/or hypercapnia to reduce metabolic activity and prevent the development of insects. However, storage pests that can tolerate hypoxia pose a challenge for long-term grain storage. More than 50% of red flour beetles can survive under hypoxia (0.5% oxygen) for eight days [24]. Several tortricid species in grain feeding can survive more than 120 days in hypoxic environments [25]. When subjected to hypoxia, *Tenebrio molitor* displays slower growth rate, higher mortality, more molting, and higher percentage of females to males than those under normoxic condition [26, 27]. Given that highly efficient RNAi technology and powerful molecular tools are widely used with *T*. *castaneum* larvae, it was particularly appealing to use a high-throughput RNA-seq method to perform global transcriptome analysis of *T*. *castaneum* larvae in response to hypoxia. The RNA-seq results allowed us to identify and functionally annotate a set of hypoxia response genes. Differences in crucial differentially expressed transcripts were validated using quantitative real-time PCR (qRT-PCR). We found that larvae under hypoxic stress enhanced glycolysis and repressed mitochondrial aerobic respiration. Our analysis of the activity of mitochondrial citrate synthase showed that hypoxia resulted in decreased enzymatic activity. The altered mitochondrial function of larvae under hypoxia may affect metabolism, leading to mitochondrial autophagy. In addition, the hypoxia-reperfusion response increased oxidative stress and levels of antioxidative enzymes, including superoxide dismutase (SOD), catalase (CAT), and glutathione-S-transferase (GST). We suggested that oxidative stress induced by reoxygenation can be eliminated by increased antioxidant enzymes in insects. The results from our study will enrich understandings of how beetle pests cope with hypoxic stress and shed light on how to control storage pests in the future.

## Materials and methods

### Insect culture and hypoxia/hypercapnia treatment

The laboratory culture of *T*. *castaneum* (Coleoptera: Tenebrionidae) was kindly donated by the Chinese Academy of Sciences. Insects were reared on whole-grain flour with 5% (w/v) yeast (OXOID, England) under dry and dark conditions (50 ± 2% relative humidity) at 30°C. About 200 adults (three days old) laid eggs in flour for 1 h, and the eggs were collected and transferred to a new diet after separation. Full-grown late-instar larvae (≥ 5 mm in length, 20 days from oviposition) were used in this research. For the hypoxia/hypercapnia treatment, 20 naked last-instar larvae were placed together in a 1-L glass bottle with whole-grain flour with 5% (w/v) yeast. Septum bottles with larvae were connected to the outlet of pressure gauges of gas cylinders via plastic tubing. Premixed gas (2% O_2_ + 18% CO_2_ + 80% N_2_; 10% O_2_ + 10% CO_2_ + 80% N_2_, Hesheng Specialty Gases, Shanghai, China) was delivered to the bottles for 9 s at 70 kPa as measured by a manometer and controlled by a regulator. Then the bottles were immediately tightly sealed with a rubber septum. A headspace analyzer (Checkpoint; Dansensor A/S, Ringsted, Denmark) analyzed the concentrations of O_2_ and CO_2_ inside the bottles to be 2.5 ± 0.2%, 18.0 ± 0.5% and 10.5 ± 0.5%, 10.0 ± 1%, respectively. Twenty control larvae were also transferred into a 1-L glass bottle with whole-grain flour with 5% (w/v) yeast, but the septum was replaced with a cotton ball to allow atmospheric air to diffuse, which maintained concentrations of 20.80 ± 0.02% oxygen and 0 ± 0.00% carbon dioxide.

For assessment of effect of hypoxia/hypercapnia on survival rate of *T*. *castaneum* larvae. After 12, 24, 36, 48, 96 h treatment, the larvae were then incubated under normoxic condition, i.e. ambient air. Three biological repeats of the hypoxic and normoxic groups were performed independently. The larvae were considered dead if they were immobile after being stimulated by sterile needle. Survival rate under various oxygen concentrations of different exposure time were calculated relative to control. One-way analysis of variance (ANOVA) followed by Tukey’s multiple range test (*P* < 0.05) was used for pairwise comparisons of the differences between treatments. To prepare RNA-seq samples, larvae were collected after 12 h of hypoxia/hypercapnia (2% O_2_ + 18% CO_2_ + 80% N_2_) treatment or normoxia treatment, and all samples were immediately frozen in liquid nitrogen for preparation of RNA-seq and qRT-PCR samples.

### RNA extraction, cDNA library preparation, sequence assembly, and annotation

Total instar RNA was extracted by using Trizol reagent (Invitrogen, Carlsbad, CA, USA) and measured the quantity by a NanoDrop spectrophotometer (NanoDrop Technologies). We confirmed the integrity of the RNA integrity by running samples on 1.5% (w/v) agarose gels. Poly (A) mRNA was extracted from total RNA using biotin-oligo (dT) magnetic beads, and then it was sheared into small pieces using an RNA fragmentation kit according to the manufacturer’s instructions (Illumina, San Diego, CA, USA). Random primers and reverse transcriptase were used to synthesize first-strand cDNA. This was followed by second-strand cDNA synthesis using DNA Polymerase I and RNase H. These fragments went through an end repair process, the addition of a single A base, and then ligation of the adapters. The ligation products were purified and size-selected by agarose gel electrophoresis. Size-selected fragments were enriched by PCR amplification. The resulting sample library was again purified and size-selected by agarose gel electrophoresis. Then we quantified the final purified products prior to seeding clusters on a flow cell. Equal volumes of hypoxia treatment and normoxia treatment samples were pooled (separately) after cDNA concentrations were adjusted to 10 nM. cDNA sequencing was conducted on a NextSeq 500 platform (Illumina) from Shanghai Personal Biotechnology (Shanghai, China).

High-quality reads were obtained from raw RNA-seq data after the removal of adapter sequences, 5′ and 3′ ends, low-quality bases (Q < 20), and < 25 bp reads. Selected reads were assembled using Trinity (Trinity Software, Plymouth, NH, USA; http://trinitynaseq.sf.net) and clustered with TGICL Clustering tools (Institute for Genomic Research, Rockville, MD, USA). The publicly available databases, NCBI non-redundant (Nr), and Kyoto Encyclopedia of Genes and Genomes (KEGG) were used to perform BLAST analysis to annotate the functions of the assembled unigenes (E cutoff = 10^−5^). The Blast2GO program (http://www.geneontology.org) was used to assign Gene Ontology (GO) annotations to the unigenes.

### qRT-PCR

Genes that were differentially expressed between the normoxia and hypoxia groups were identified based on fragments per kilobase per million mapped reads (FPKM) value, which converted the number of fragments mapped to a transcript according to the total number of fragments mapped to all unigenes and the length of the transcript. The DEseq software was used to analyze count data of the RNA-seq and test for the differential expression of genes. First, the high-quality reads were mapped to unigenes to calculate the number of reads mapped to each unigene. Then the raw read counts were input DEseq software to get the normalized signal for each unigene, and the fold change of unigene expression values. A value of *p* ≤ 0.05, FDR ≤ 0.001 and fold change ≥1.5 provided significance thresholds for gene expression differences. Nine differentially expressed genes in vital hypoxia response pathways with good annotation were selected to validate RNA-seq data using qRT-PCR. The RNA samples were the same ones used for RNA-seq mentioned previously. To prepare qRT-PCR templates, we used 2 μg of RNA to synthesize cDNA with random primers and M-Mulv reverse transcriptase (New England Biolabs, Beverly, MA, USA). qRT-PCR reactions were conducted using Power SYBR Green PCR Master Mix and run on a real-time thermal cycler (Bio-Rad, USA). Briefly, each reaction volume was 20 μL, including 10 μL of Premix Ex Taq, 4 μL of each primer (1 mM), and 2 μL of cDNA (1/100 dilution). The qRT-PCR reaction proceeded at 95°C for 5 min; this was followed by 40 cycles of 95°C for 10 s, 60°C for 20 s, 72°C for 20 s, and 72°C for 5 min; the final step was a melting curve analysis achieved by gradually heating the product from 60°C to 95°C. 18s rRNA was used as the internal reference [28]. The primers for analyzed genes are listed in Table 1. Expression levels were calculated using the comparative C_T_ method. Gene expression patterns were analyzed using the 2^–ΔΔCT^ approach.

**Table 1:** The primers of analyzed genes by qRT-PCR.

Gene	Forward primer 5’-3’	Reverse primer 5’-3’
18S rRNA	ATGGTTGCAAAGCTGAAACT	TCCCGTGTTGAGTCAAATTA
Elongation of very long chain fatty acids protein 7	GCGGGTGGTTCTGGGATT	GGGCGTGAGGCTGTGATG
ATP-binding cassette subfamily C	AAAAGTGTCCAAGAAAGT	CACCCAGAAAGTAATAAA
Heat shock 70kDa protein	ACGAAAATGAAGGAAACC	GTCAAGCCCATAAGCCAA
MAP kinase	GCGGTGGTGGAGGTTCTG	GTGCGTTTCTTTTTGCGG
Dual specificity MAP kinase phosphatase	GTGCTGCCCTTCTTGTAT	GAGGTTTTGATGTCCGTT
Superoxide dismutase	CCATTTCAACCCCTACCT	CACACTGCCATCTTCCTC
Four-jointed box protein 1	GCTTTGTATTCGGTGGAG	TGTATGGAAATCTTGCGT
FJBP	GCAGTTGCGCGTTTTCGT	AGCCCACTCTCGTTGTCC
Alpha-glucosidase	GGTTACAACGATACAGAT	CACTACCATAATAGAGCA
Hexokinase	TGCTCAAAGATGCGATTGAC	GTCAAACATTTCGGCGTTTT
Phosphoglucose isomerase	GTATTCCCTGTGGTCCGCTA	CTGGTCGTAGGGCAAGAGAG
6-phosphofructo-2-kinase	TGCGGTCGATACTCACAGAG	GGCACCCTGTATCAGTCGAT
Fructose 1,6-bisphosphate aldolase	ATCAAGGACCACACTCCCAG	TCTGTGACTTTCTGGCAACG
Glyceraldehyde-3-phosphate dehydrogenase	CTTCAAGGGCGAAGTCAAAG	AGGCTTTTTCAATGGTGGTG
Phosphoglycerate kinase	GAAGCAAAACAACGTGCAAA	GCCCGTTCCACACTATCACT
Phosphoglycerate mutase	GCAAATCTCAGCGAAAAAGG	GGGCGCGTGTTAATACTGAT
Enolase	AGCCGCCTCTGAGTTTTACA	GAAGGGATCCTCAATGGACA
Pyruvate kinase	GTAAAGCTGCAGAAGGTGCC	CCCGTCGTCGTAATCAGAAT
L-lactate dehydrogenase	TGCTTATTGACTGCGTGGAG	TTGGACTAAATCCAGACGGG
Pyruvate dehydrogenase	CCGCACTCATGAAGAAGTCA	TTGCACTTGGCTACAGCATC
Aconitate hydratase	GGCACGGTGGACATAGACTT	CCAACTTGACGAACCCAACT
Isocitrate dehydrogenase	GACCAACACCACTGTCAACG	AGGATGTCGACAACAGGTCC
Succinyl-CoA synthetase	CCCAAGTCGGTTTAGGTCAA	CGCATTAGGCCCAGTATTGT
Succinate dehydrogenase	GTTCAAGGTGGCCAAAGGTA	GGCATTCAAATCGACCTTGT
Fumarate hydratase	GTGGAAATCCAAAACACGCT	CGAACTTCATCTGCTGGACA
Malate dehydrogenase	CCCACCAATTACGCAAGTCT	ACGTCGACGGTAGTTATGCC

### Measurement of the activity of detoxification enzymes and oxidative damage

To prepare the samples, we subjected new batches of larvae to 12 h of hypoxia treatment. The hypoxia groups were then returned to normoxia for recovery. Twenty larvae were collected at 0, 6, 12, or 24 h and used as the treatment group. And twenty untreated larvae from the same batch larvae were used as the control groups. The activities of SOD, CAT, and GST activities and malondialdehyde (MDA) concentrations were determined in both treated and untreated groups. Three biological replicates were performed. To prepare the protein samples, we homogenized the larvae in 1 mL of ice-cold PBS and centrifuged at 10,000 *g* for 15 min at 4°C. The supernatant was used for protein quantification by Bradford assays.

SOD activity was determined as the suppression of the reduction rate of nitroblue tetrazolium (NBT). In brief, an 80 μL sample was mixed with 20 μL of xanthine oxidase solution (10 mg bovine albumin, 100 μL xanthine oxidase dissolved in 2 mL PBS) and 500 μL of reaction solution (70 μM NBT, 125 μM xanthine). The mixture was monitored at an absorbance of 560 nm for 15 min using an Epoch Microplate spectroscopy system (BioTek Instruments, Winooski, VT, USA). Three replicates were performed. SOD activity was presented as the difference in absorbance between the mixture and the blank.

CAT activity was estimated as the decomposition rate of hydrogen peroxide (H_2_O_2_) [29]. Samples were diluted 100 times with PB solution (0.05 M, pH 7.5). Then 1 mL diluted samples were added into quartz cuvettes and mixed with 500 μL of 1% H_2_O_2_. The mixture was placed on a spectrophotometer and a record made every 30 s for 10 min at an absorbance of 240 nm. The resultant absorbance plot displayed an exponential decay curve. Three replicates were performed. CAT activity was calculated using the following formulas: k = 1/300 × (lnA_3_/A_8_) (A_3_ = absorbance at 3 min, A_8_ = absorbance at 8 min), k_total/mL_ = k/sample volume, and k/mg = k_total/mL/mg_ protein.

GST activity was determined as the concentration of 5-(2,4-dinitrophenyl)-glutathione catalyzed by GST. Protein samples were diluted with sodium phosphate buffer (0.1 M, pH 6.5) and a 100 μL sample was added to a 96 well plate. Then 100 μL of GSH (12 mM) and 100 of μL 2,4-dinitrochlorobenzene (DNCB, 1.2 mM) were added. The mixture was immediately measured at 340 nm using an Epoch Microplate spectroscopy system (BioTek Instruments, Winooski, VT, USA). Three replicates were performed. GST activity was expressed as ΔAbsorbance at 340 nm/min/mg protein.

For lipid peroxidation activity, samples were collected 1 d and 5 d after hypoxia-reperfusion. The aim was to detect the level of reperfusion damage and recovery in larvae. The concentration of MDA was used to measure lipid peroxidation reactions [30]. Supernatant (250 μL) was mixed with 125 μL of 20% (v/v) trichloroacetic acid (Sigma). After centrifugation at 15,000 *g* for 10 min at 4°C, 300 μL of supernatant was mixed with 300 μL of 0.8% 2-thiobarbituric acid (w/v) reagent. The mixture was incubated in a 100°C water bath for 60 min and cooled on ice for 5 min. Then it was centrifuged at 4,000 *g* for 1 min at room temperature. The absorbance of chromophore was measured at 535 nm. Three replicates were performed. The nmol MDA produced per milligram protein was calculated based on a molar extinction coefficient of 1.56 × 10^5^ M^–1^cm^–1^.

All data analyses were performed using SPSS version 20 software (SPSS, Chicago, IL, USA). Bars represent the mean ± SE. One-way analysis of variance (ANOVA) followed by Tukey’s multiple range test (*P* < 0.05) was used for pairwise comparisons of the differences between treatments.

### Mitochondria isolation and citrate synthase assay

Larvae were subjected to hypoxic stress for 12 h and returned to normoxia to allow them to recover. Then larval samples were collected at 0, 6, or 12 h during recovery. The mitochondria isolation assay was performed as previously described [31]. In brief, a number of 100 larvae were collected and homogenized in 1 mL of ice-cold mitochondria isolation buffer (200 mM sucrose, 10 mM MOPS, 1 mM EGTA, pH 7.4). The supernatant was transferred to a centrifuge tube with 10 mL of ice-cold fresh mitochondria isolation buffer. After centrifugation at 600 *g* for 10 min at 4°C, the pellet was discarded. The supernatant was centrifuged at 7,000 *g* for 10 min at 4°C, and the pellet was washed with 5 mL of ice-cold mitochondria isolation buffer. The pellet represented a sample of mitochondria. The biuret test was used to determine the concentration of mitochondrial protein before storage at –80°C. Prior to the assay, the samples were subjected to three freeze-thaw cycles using liquid nitrogen to break the mitochondrial membranes.

For the citrate synthase activity assay, it was measured as previously described [32]. Briefly, we measured citrate synthase by monitoring the reduction in dithiobis nitrobenzoic acid (DTNB; colorless) to thionitrobenzic acid (yellow) at 412 nm. Mitochondria samples (~0.5 μg) were incubated in 200 μL of reaction mixture (2 mM DTNB, 0.1 mM acetyl CoA, 1 mM oxaloacetic acid) in a 96 well plate. The plate was placed on an Epoch Microplate spectroscopy system (BioTek Instruments). We expressed citrate synthase activity as nmol/min/ng protein using a molar extinction coefficient of 1.36 × 10^4^ M^–1^cm^–1^. Data from different groups were analyzed by one-way ANOVA. Tukey’s multiple test was used for pairwise comparisons of differences between treatments for mean separation (*P* < 0.05).

## Results and discussion

### The influence of hypoxia/hypercapnia on survival rate, behavioral reactions and morphology of insects

**Fig 1:**
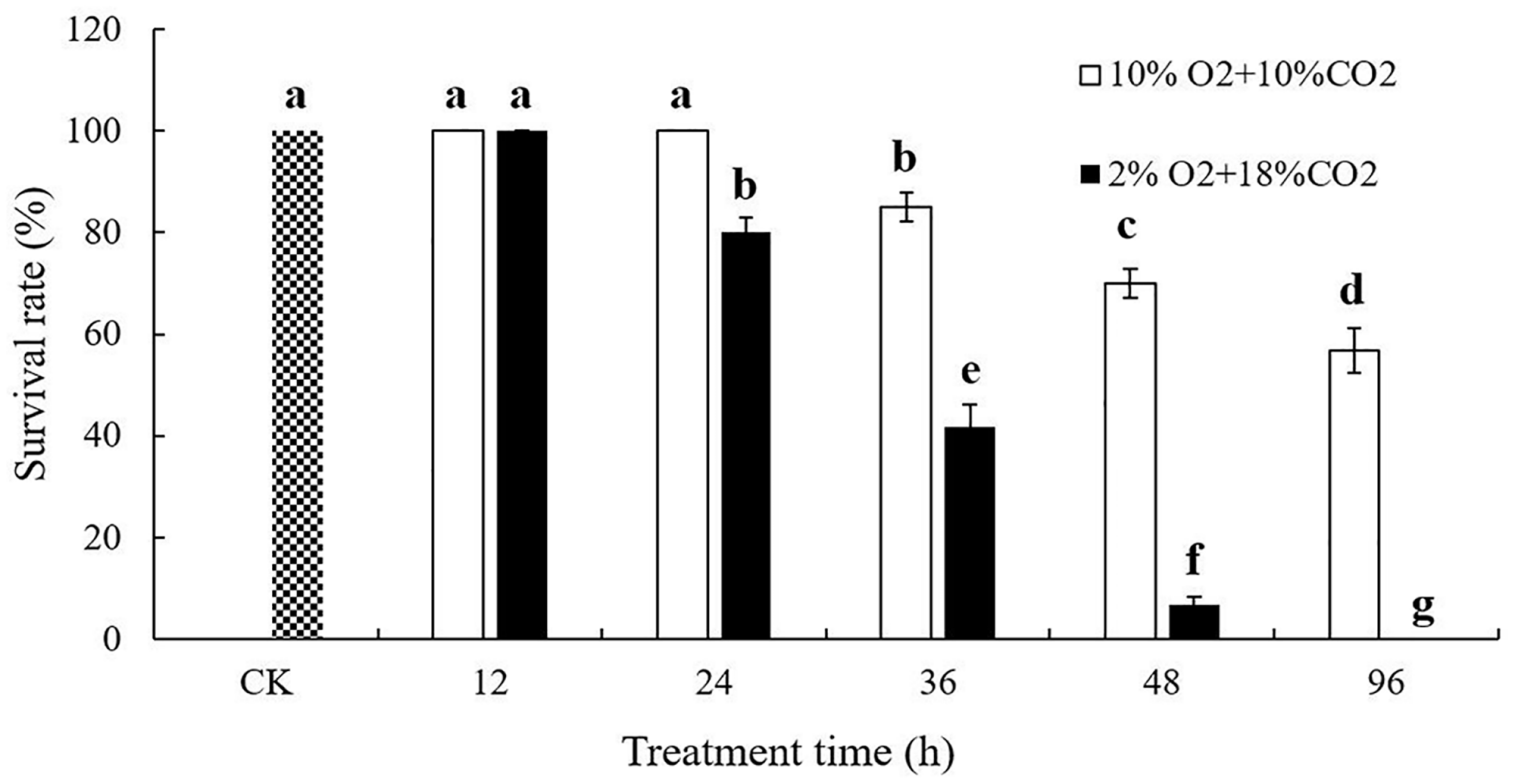
The influence of hypoxia/hypercapnia on survival rate of *T*. *castaneum* larvae. The survival rate of *T*. *castaneum* larvae, which were exposed to 2% O_2_ + 18% CO_2_ + 80% N_2_ or 10% O_2_ + 10% CO_2_ + 80% N_2_, were analyzed. Insects grow under normoxia were used as the control. Shown are the mean and SE of three biological replicates. Different letter types indicate significant differences (*P* < 0.05) by Tukey’s multiple range test.

Organisms have evolved special strategies to resist environmental stresses due to hypoxia and thus remain alive [1, 33]. When *T*. *castaneum* larvae was exposed to 2% O_2_ + 18% CO_2_ + 80% N_2_ with 24, 36 and 48 h, the survival rate was 80%, 41% and 6%, respectively, indicating that lower oxygen concentration (2% O_2_ + 18% CO_2_ + 80% N_2_) resulted in a more severe damage to *T*. *castaneum* larvae than 10% O_2_ + 10% CO_2_ + 80% N_2_ (Fig 1). A relatively short-term (e.g. 12 h) hypoxia/hypercapnia (2% O_2_ + 18% CO_2_ + 80% N_2_) can not result in visible detrimental effects on *T*. *castaneum* larvae. However, exposure of 96 h lead to 100% mortality of *T*. *castaneum* larvae under hypoxia/hypercapnia. Compared to the control group under normoxia, the hypoxia-treated larvae appeared to stop moving and feeding. Our observation suggested that the larvae might reduce movement under hypoxic stress to conserve energy. Relative to normoxic environment, the midgut of cowpea bruchid larvae (*Callosobruchus maculatus*) became shrunken on size under hypoxia [34]. Bruchid larvae decrease assimilative activity under hypoxia to achieve the balance of ATP supply and consumption considering gut proteases are required to break down dietary proteins, which are the major energy sinks under normoxia in insects. Insects of high altitude are likely to have reduced the capability of flight and shrunken the size of wings, which causing reduced requirements for O_2_ and they may be adaptations to hypoxic environment [35]. Previous findings indicate that hypoxia suppresses mitochondrial activities and activates dysfunctional proteins clearance in endoplasmic reticulum based on genome-wide analysis in thoracic muscle of the migratory locust [36]. Besides, hypoxia tolerant insects might enlarge the area of tracheal cross-sections to cope with low oxygen stress, thereby allowing a greater volume of air to reach tissues. For example, the hypoxic environment under 15% and 10.5% oxygen could induce an increase to 40% and 120% respectively in tracheal cross-sections volume in yellow mealworms [37]. It is reported that American locust (*Schistocerca americana*) becomes quiescent and ventilatory frequency and tracheal conductance increased under hypoxic stress [38]. Damsel fly nymphs increase their rate of ventilatory movement in hypoxic water [39]. Behavioral changes enable aquatic insects to increase the amount of oxygen available in response to hypoxia [40]. Hypoxia also affected the growth rate of larvae, relative to normoxic ones. The developmental abnormalities at ecdysis and a female-biased sex ratio are observed in *T*. *molitor* larvae [41]. Recently, it was reported that hypoxia might have effect on honey bee (*Apis mellifera*) queen/worker development. Queen larvae have more mitochondrial function units in their fat body, indicating they have higher energy production capacities and lead to different caste development [42].

It is interesting that *T*. *castaneum* larvae can recover from hypoxic stress when returned to normoxia (Fig 1), which is consistent with many previous reports on insects, including grain weevil (*Sitophilus granarius*), cerambicid larvae (*Orthosoma brunnem*), and desert locust (*Schistocerca gregaria*) [40]. A *Drosophila* line obtained from hypoxia selection showed a reduced body size and weight. Hypoxia-selected fly embryos recovered to a normal size from a shrunken size, once returned to normoxia [14, 43, 44]. Our results showed that 12 h hypoxia/hypercapnia treatment can hardly damage *T*. *castaneum* larvae, which suggested that some hypoxia response genes may be induced to cope with oxygen deprivation. Then we used the high throughput sequencing tools to identify hypoxia response genes and investigate hypoxia resistance mechanisms of insects.

### Analysis of RNA-seq data and validation of differentially expressed genes (DEGs) using qRT-PCR

#### Illumina sequence analysis and *de novo* assembly

Totals of 17,307,627 and 19,866,284 paired-end raw reads were generated from the control and hypoxia groups, respectively, using a NextSeq 500 platform (Illumina). Totals of 12,524,554 useful reads from the control group and 14,193,339 useful reads from the treatment group were generated after Q20 filtering. GC contents were 48.09% and 50.74%, respectively (S1 Table). To create RNA-seq maps, we compared these useful reads to the *T*. *castaneum* genome sequence (http://www.beetlebase.org). A total of 16,524 unigenes had been annotated. In this study, DEGs were defined as genes exhibiting a fold transcriptional change ≥1.5 with *p* ≤ 0.05 and FDR ≤ 0.001 (S1 Fig and S2 Table). Of the 333 identified DEGs, 154 were downregulated and 179 were upregulated. Some DEGs involved in hypoxia response pathways are shown in Table 2.

**Table 2:** Some hypoxia-response DEGs in the RNA-seq.

Functional description	Gene names	Genes ID	Foldchange	Chromosome	Length	References
Glycolysis	Hexokinase	TC000319	1.86	ChLG2	6043	[22, 61, 81, 82]
Glycolysis	Glycerol-3-phosphate dehydrogenase	TC014609	2.32	ChLG5	5150	
Glycolysis	Enolase	TC011730	1.93	ChLG9	1431	
Glycolysis	Pyruvate kinase	TC006543	-1.95	ChLG8	1608	
Kreb’s cycle	Aconitate hydratase	TC010417	-1.67	ChLG5	9486	
anaerobic metabolism	L-lactate dehydrogenase	TC003922	2.35	ChLG3	10038	
Glucose transporter	Glucose transporter member 1	TC013486	2.40	ChLG5	1847	[61, 83]
Mitochondrial mitophagy	BNIP3; BCL2/adenovirus E1B 19 kDa protein-interacting protein 3	TC010884	1.84	ChLGX	808	[61, 64, 65]
	Autophagy-related protein 13	TC001577	2.11	ChLG3	4744	
Hippo signaling pathway	DACHS; dachs	TC010547	-2.88	ChLG3	90137	[46, 84]
	Four-jointed box protein 1	TC015492	-2.36	ChLG6	1191	
MAPK signaling pathway	MAP kinase	TC010495	2.02	ChLG3	39394	[85]
	Dual specificity MAP kinase phosphatase	TC010766	1.74	ChLGX	7861	
Hedgehog signaling pathway	Growth arrest-specific 1	TC009285	2.98	ChLG7	2969	[86]
Peroxisome	Superoxide dismutase	TC011675	2.00	ChLG9	927	[87]
Signaling transduction	Ras homolog gene family	TC009159	1.70	ChLG7	579	[88]
Signaling transduction	Sodium/potassium/chloride transporter member 2	TC000573	1.93	ChLG2	6881	[89, 90]
Metabolism	Heat shock 70kDa protein	TC010172	2.16	ChLG7	1941	[50, 91]

#### Functional annotation and classification of the hypoxia transcriptome in *T. castaneum*

**Fig 2:**
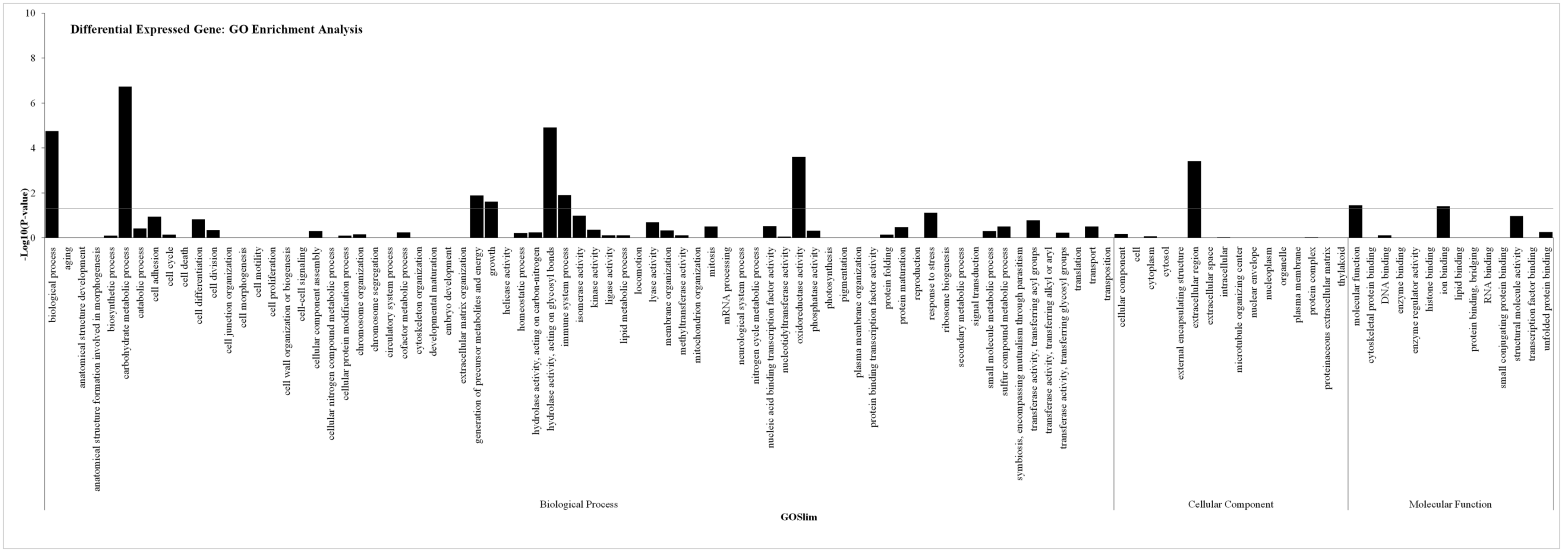
GO enrichment analysis of genes involves in the hypoxia resistance. The GO terms are sorted by —Log 10 of the enrichment *P*-value, which represent the enrichment significance of GO terms. The enrichment of GO terms are shown by comparing DEG with the whole genome.

In this study, we divided the identified genes into 62 GO functional subcategories. We found that many DEGs were involved in carbohydrate metabolism, hydrolyzed glycosyl bonds, and oxidoreductase activity (Fig 2). Other GO terms that were enriched included generation of precursor metabolites; energy, growth, and immune system processes; molecular function; and ion binding (Fig 2). In total, 333 DEGs were classified into six principal KEGG categories containing 42 secondary pathways (S2 Fig). Metabolism was the largest category (110, 40.44%), followed by human diseases (56, 20.59%), organismal systems (45, 16.54%), cellular processes (27, 9.93%), environmental information processing (24, 8.82%), and genetic information processing (10, 3.68%). These results indicate that both metabolic and genetic processes are involved in responses to hypoxia in larvae.

#### Validation of differential transcript expression using qRT-PCR

**Fig 3:**
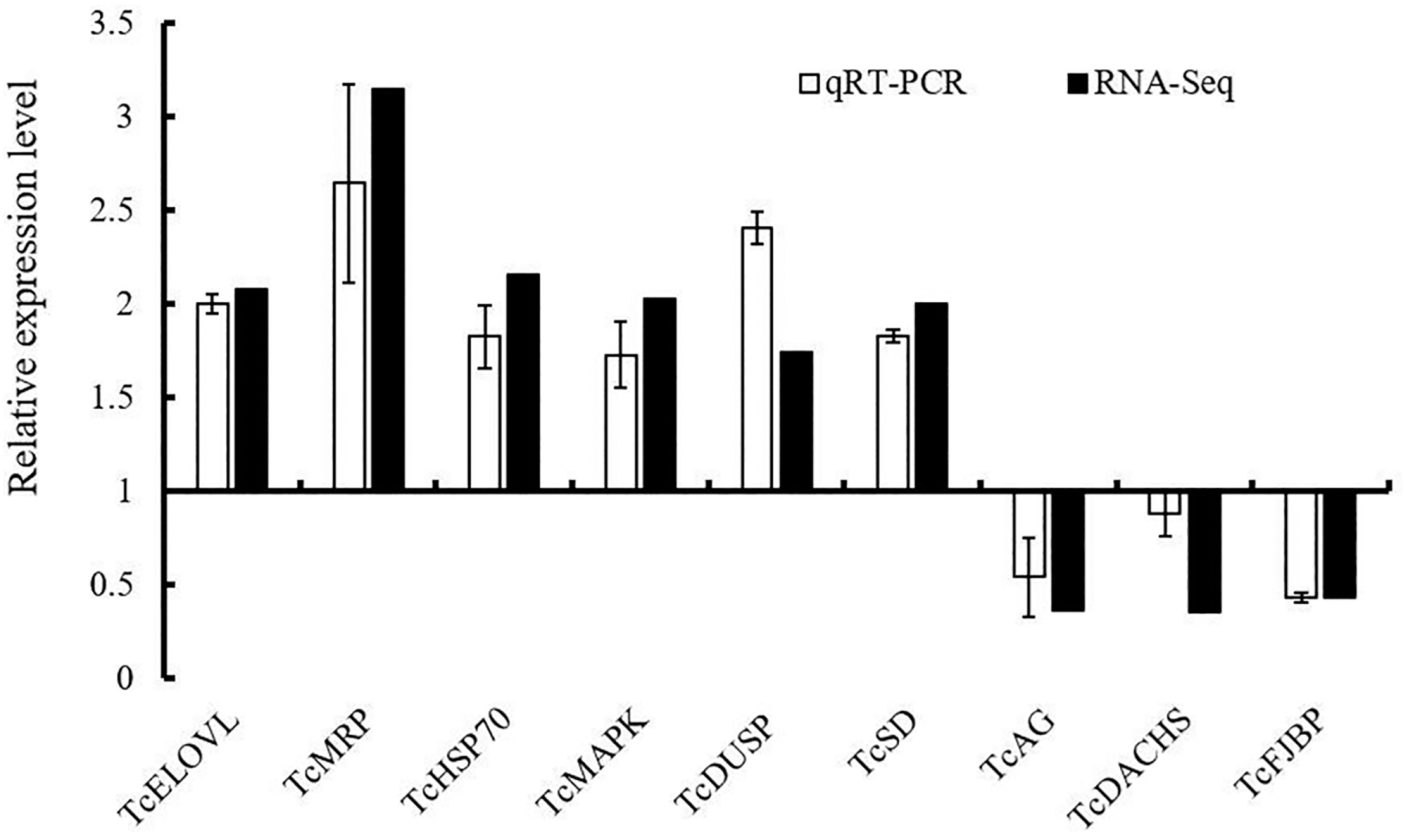
qRT-PCR analysis of selected transcripts to confirm expression profiles identified by RNA-seq. *Tc*ELOVL, elongation of very long chain fatty acids protein 7; *Tc*MRP, ATP-binding cassette subfamily C (CFTR/MRP) member 4; *Tc*HSP70, heat shock 70kDa protein; *Tc*MAPK, MAP kinase; *Tc*DUSP, Dual specificity MAP kinase phosphatase; *Tc*SD, superoxide dismutase, Cu-Zn family; *Tc*AG, alpha-glucosidase; *Tc*DACHS, DACHS, Hippo signling pathway; *Tc*FJBP Four-jointed box protein 1 (FJBP). Value represents mean ± SE of three independent PCR amplifications and quantifications.

To verify the gene expression profiles identified by RNA-seq, we used qRT-PCR to measure the expression of nine selected genes. These genes encoded elongation of very-long-chain fatty acids (*Tc*ELOVL), the ATP-binding cassette subfamily (*Tc*MRP), heat shock 70 kDa protein (*Tc*HSP70), MAP kinase (*Tc*MAPK), dual-specificity MAP kinase phosphatase (*Tc*DUSP), Cu-Zn superoxide dismutase (*Tc*SD), alpha-glucosidase (*Tc*AG), DACHS (*Tc*DACHS), and four-jointed box protein 1 (*Tc*FJBP). In general, the qRT-PCR data matched the RNA-seq results well (Fig 3). *Tc*ELOVL, *Tc*MRP, *Tc*HSP70, *Tc*MAPK, *Tc*DUSP, and *Tc*SD were upregulated, whereas *Tc*AG, *Tc*DACHS, and *Tc*FJBP were downregulated, as shown by both qRT-PCR and RNA-seq.

### Hypoxia affected several signaling transduction pathways of *T*. *castaneum*

Insects under hypoxic stress temporarily regulate gene transcription and protein expression to overcome this stress. Previous research showed that the nitric oxide/cyclic GMP pathway might be activated in response to hypoxia to regulate insects’ survival, tracheal development, and cell division [45]. Recently, it is shown in *Drosophila* that Hippo tumor-suppression pathways, regulated growth under hypoxia [46]. In our study, *Tc*FJBP (TC015492) and DACHS (TC010547), both of which are involved in the Hippo signaling pathway, were downregulated in response to hypoxia (Fig 3), which suggested that the Hippo pathway might affect oxygen deprivation and that this may in turn suppressed cell and tissue growth. However, the relationship between hypoxia response behavior and signaling pathway is largely unknown in coleopterans and needs further investigation in the future.

Several genes involved in signaling transduction pathways were also identified by our RNA-seq analysis (Table 2). For example, two MAPK signal pathway genes (MAP kinase, TC010495; dual specificity MAP kinase phosphatase, TC010766) were induced by hypoxia. Hypoxia may alter intracellular Ca^2+^ levels, regulate the MAPK cascade, dephosphorylate key protein kinases, and determine cellular apoptosis or survival [47]. It is noteworthy that a tyrosine 3-monooxygenase (TC002496) was upregulated in *T*. *castaneum* under hypoxia. Given that the tyrosine 3-monooxygenase or tyrosine hydroxylase is the rate-limiting enzyme in the biosynthesis of dopamine, it might have a vital function in regulating cellular respiratory adaptation to hypoxia [48, 49].

Molecular chaperones, mainly the heat shock protein family, might have some function in organisms under hypoxic stress. Heat shock protein 70 (Hsp70) plays a protective role and improves survival of insects under hypoxic conditions. Overexpression of Hsp70 markedly improves the survival rate under conditions of severe hypoxia in *Drosophila* [50]. In our study, results of both qRT-PCR and RNA-seq indicated that Hsp70 (TC010172) was upregulated under hypoxia relative to the control group (Fig 3). Such upregulation of Hsp70 suggests that it might be involved in protecting larvae by preventing protein aggregation under hypoxia [50].

### Hypoxia-induced glycolysis and suppressed mitochondrial aerobic respiration for insects

**Fig 4:**
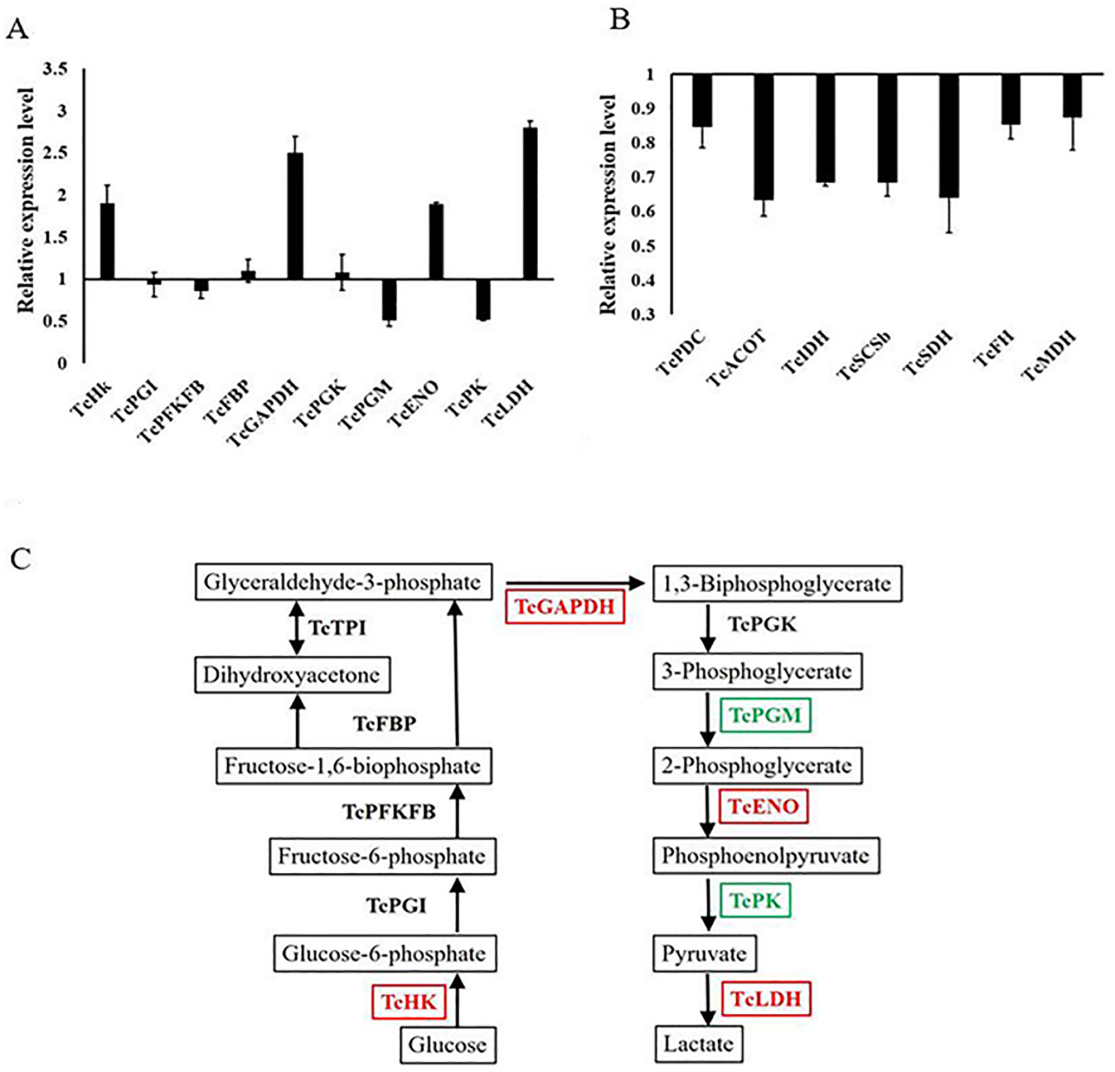
Gene expression pattern of glycolytic (A) and Krebs (B) cycle enzymes of *Tribolium castaneum* larvae in response to hypoxia. Total RNA was isolated from the larvae after 12 hours’ hypoxia treatment. qRT-PCR was used to illustrate gene expression. *Tc*HK, hexokinase; *Tc*PGI, phosphoglucose isomerase; *Tc*PFKFB, 6-phosphofructo-2-kinase/fructose-2,6-bisphosphatase; *Tc*FBP, fructose 1,6-bisphosphate aldolase; *Tc*TPI, triosephosphate isomerase; *Tc*GAPDH, glyceraldehyde-3-phosphate dehydrogenase; *Tc*PGK, phosphoglycerate kinase; *Tc*PGM, phosphoglycerate mutase; *Tc*ENO, Enolase; *Tc*PK, Pyruvate kinase; *Tc*LDH, L-lactate dehydrogenase; *Tc*PDC, pyruvate dehydrogenase; *Tc*ACO, aconitate hydratase, mitochondria; *Tc*IDH, isocitrate dehydrogenase; *Tc*SCSb, succinyl-CoA synthetase beta chain; *Tc*SDH, succinate dehydrogenase; *Tc*FH, fumarate hydratase; *Tc*MDH, malate dehydrogenase. Red color represents upregulate, green color represents downregulate and black color represents no change.

The hypoxic transcriptional results identified some changes in certain glycolytic genes. The expression profiles of 10 genes encoding enzymes involved in glycolysis and seven genes encoding enzymes involved in the Krebs cycle were assessed by qRT-PCR (Fig 4). The results suggested that cellular metabolism shifted from aerobic respiration to anaerobic metabolism. Both RNA-seq and qRT-PCR results showed that hexokinase (TC000319), enolase (TC011730), and glycerol-3-phosphate dehydrogenase (TC014609) were upregulated under hypoxia (Fig 4). Other glycolytic enzymes, such as *Tc*PGI, *Tc*PFKFB, *Tc*FBP, and *Tc*PGK, showed no obvious difference between the control group and the treatment group. It was interesting that glycerol-3-phosphate dehydrogenase facilitates mitochondrial bioenergetics capacities, is involved in mitochondrial O_2_^-^ production and may have functions under hypoxia [51]. Hexokinase, the rate-limiting enzyme of glycolysis, was activated to convert glucose into glucose 6-phosphate, thus promoting glycolysis. L-lactate dehydrogenase (TC003922) was upregulated more than twofold in response to hypoxia. Lactate dehydrogenase converts pyruvate to lactate and regenerates NAD^+^, a continuous supply of which is required for glycolysis. These data indicate that hypoxic *T*. *castaneum* promoted glycolysis to enhance anaerobic carbohydrate consumption in response to hypoxia. However, all enzymes involved in the Krebs cycle were downregulated (Fig 4), which suggests that insects under hypoxia suppressed mitochondrial TCA cycle enzyme expression.

Some genes encoding enzymes involved in carbohydrate metabolism were downregulated in insects under hypoxia. Expression levels of dihydrodiol dehydrogenase/D-xylose 1-dehydrogenase (NADP; TC014229), alpha-amylase (TC000936, TC000938, TC000937, TC000939), alpha-glucosidase (TC008357), beta-1,3-galactosyltransferase (TC008954), chitinase (TC012734, TC009177, TC009176), and glucuronosyltransferase (TC007056) were all reduced more than twofold. This suggests that hypoxia seems to lead to a general suppression of carbohydrate metabolism.

Aerobic insects can depress their metabolism and adjust their energy requirements in low-oxygen environments [14]. In desert locust, anoxia results in 6% metabolic depression compared to a control group [52, 53], whereas about 3% metabolic depression is documented in tiger beetle larvae [54]. In fact, the level of ATP declines while levels of ADP, IMP, and AMP increase within a few minutes of exposure to hypoxia [40]. End products of anaerobic metabolism have been detected in a few terrestrial insect species. The predominant end products of insects in anaerobic metabolism are lactate and alanine [55, 56].

Together, our results indicate that aerobic respiration of *T*. *castaneum* was restrained because of limited available oxygen. To balance ATP demands under stress due to low oxygen, the larvae increased glycolytic enzyme expression to generate pyruvic acid and preferentially convert it to lactic acid by anaerobic metabolism (Fig 4). The upregulation of certain rate-limiting enzymes, for example hexokinase and lactate dehydrogenase, required that the larvae turn to anaerobic metabolism for survival in response to low oxygen.

### Hypoxia suppressed the activity of citrate synthase and might induce mitophagy of insects

**Fig 5:**
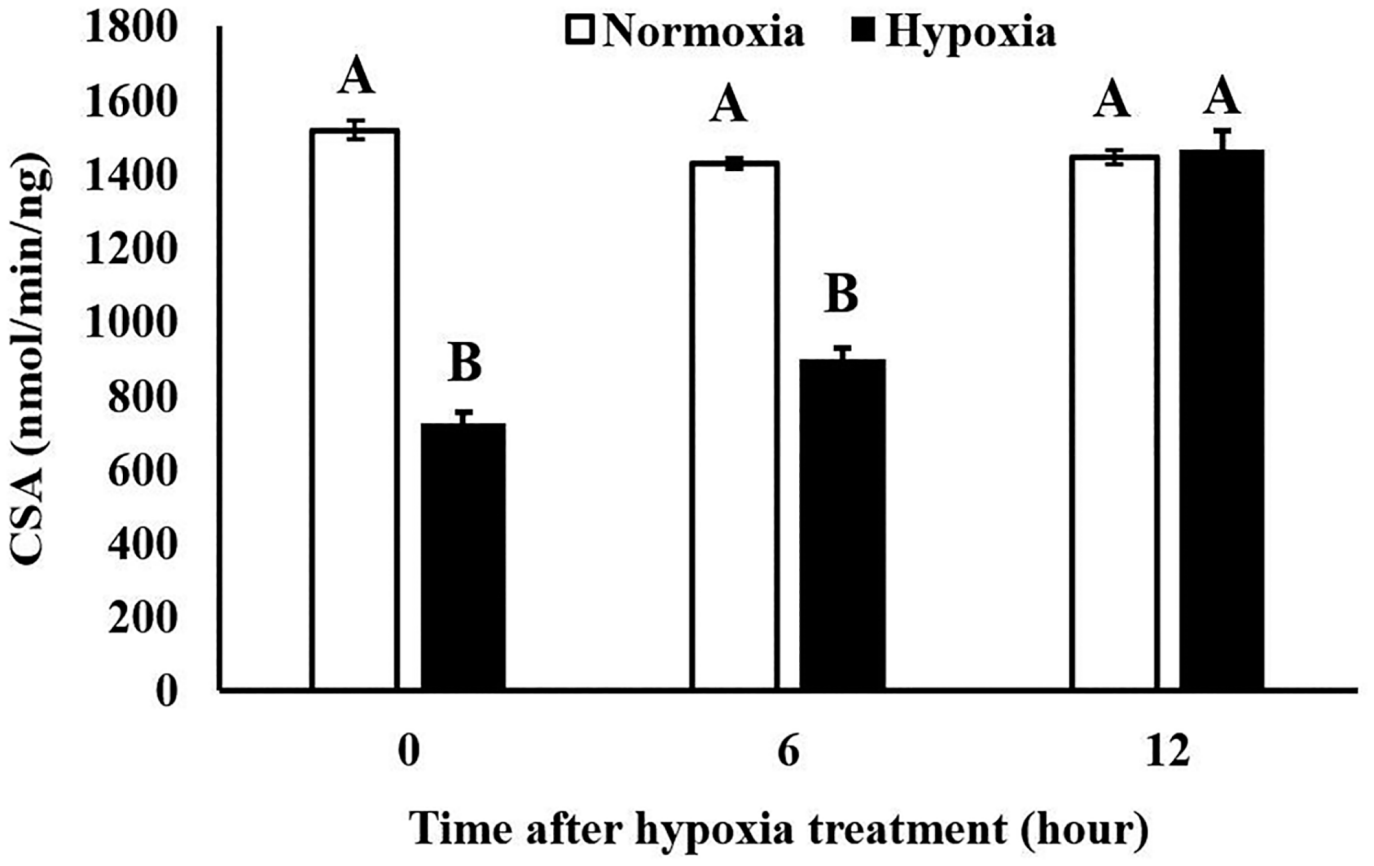
Variation in citrate synthase activity of *T*. *castaneum* under normoxia and hypoxia-reperfusion. The citrate synthase activity of different groups was analyzed by one-way ANOVA. Results are shown as mean ± SE. Tukey’s multiple test was used for pairwise comparison of the difference between treatment for mean separation (*P* < 0.05).

In our study, the activity of citrate synthase was investigated under hypoxic or normoxic conditions to assess larvae’ recovery from hypoxia (Fig 5). Compared to the control group, mitochondrial citrate synthase was significantly altered after hypoxia treatment of insects (Fig 5). It is interesting that citrate synthase in the hypoxic group recovered after 12 h of exposure to normoxia compared to 0 h and 6 h. The ability of insects to cope with this stress might be due to temporary changes in mitochondrial gene expression that facilitate survival. The enzyme citrate synthase exists in the mitochondrial matrix and functions in the first step of the citric acid cycle of all living organisms. It is usually used as a quantitative enzyme marker for the presence of mitochondria, aerobic capacity, and mitochondria density in cells [57–60]. The decreased citrate synthase under hypoxia suggests that low oxygen might suppress mitochondrial activity. The decreased citrate synthase coupled with the qRT-PCR data suggest that mitochondria were the main target in insects under hypoxia. Emerging evidence has illustrated that hypoxia suppresses mitochondrial biogenesis and decreases mitochondrial mass and oxygen consumption [61]. This type of mitochondrial remodeling in response to hypoxia includes replication of the mitochondrial genome as well as coordinated expression of nuclear and mitochondrial encoded proteins [62]. In *D*. *melanogaster*, hypoxia treatment for 90 min significantly inhibited citrate synthase and resulted in a marked reduction in life span [59]. Diminished mitochondrial aerobic capacity and oxidative phosphorylation are always associated with a reduced life span. Altered mitochondrial function could lead to a reduced life span because of accumulated damage in cell and tissue [59].

Autophagy is an evolutionarily conserved process by which organelles and macromolecules are degraded [63]. Previous reports have indicated that several outer mitochondrial membrane proteins serve as mitophagic receptors in mammals, including BNIP3 (BCL2/adenovirus E1B 19 kDa protein-interacting protein 3), FUNDC1 (FUN14 domain-containing protein 1), and BNIP3-like (BNIP3L/NIX) protein [61]. In yeast, autophagy-related (Atg) protein 8 and its mammalian homologs induced autophagy [64]. According to our RNA-seq data, two genes involved in autophagy were upregulated under hypoxia in larvae. These genes were encoded by autophagy-related (Atg) protein 13 (TC001577) and BNIP3 (TC010884). BNIP3 is a mammalian mitophagic receptor that promotes apoptosis or programmed necrosis [65]. Under hypoxic conditions, the phosphorylation of BNIP3 at serine flanking the LC3-interacting region domain promotes BNIP3 binding to members of the LC3 protein family, ultimately enhancing mitophagy [66]. Emerging evidence indicates that mitophagy is a form of selective protection against low-oxygen damage to mitochondrial metabolism [67]. Future research might focus on hypoxia-induced mitochondrial autophagy and investigate the relationship between free radical and autophagy-related genes for insects under hypoxia.

### Antioxidants were increased to protect insects from reoxygenation-induced oxidative damage

Hypoxia-reperfusion alters the cellular redox state and triggers the release of ROS [68]. The electrons that drive the mitochondrial respiration chain are reduced under hypoxia in insects. When oxygen is reintroduced, an immediate reoxidation occurs that is always associated with an overproduction of oxygen free radicals [69]. The burst in oxyradical production can damage macromolecules, including membrane lipids and DNA [70, 71]. Recently, it was reported that increased ROS level activate tyrosine kinase, phosphorylate mitochondrial complex II submit FpSDH, and adjust the level of complex I to optimize NADH/FADH_2_ electron use in response to hypoxia/reoxygenation [72]. In mammals, overexpression of antioxidative enzymes and the use of exogenous antioxidants have beneficial effects for reducing oxyradical damage caused by reoxygenation or reperfusion [73, 74].

**Fig 6:**
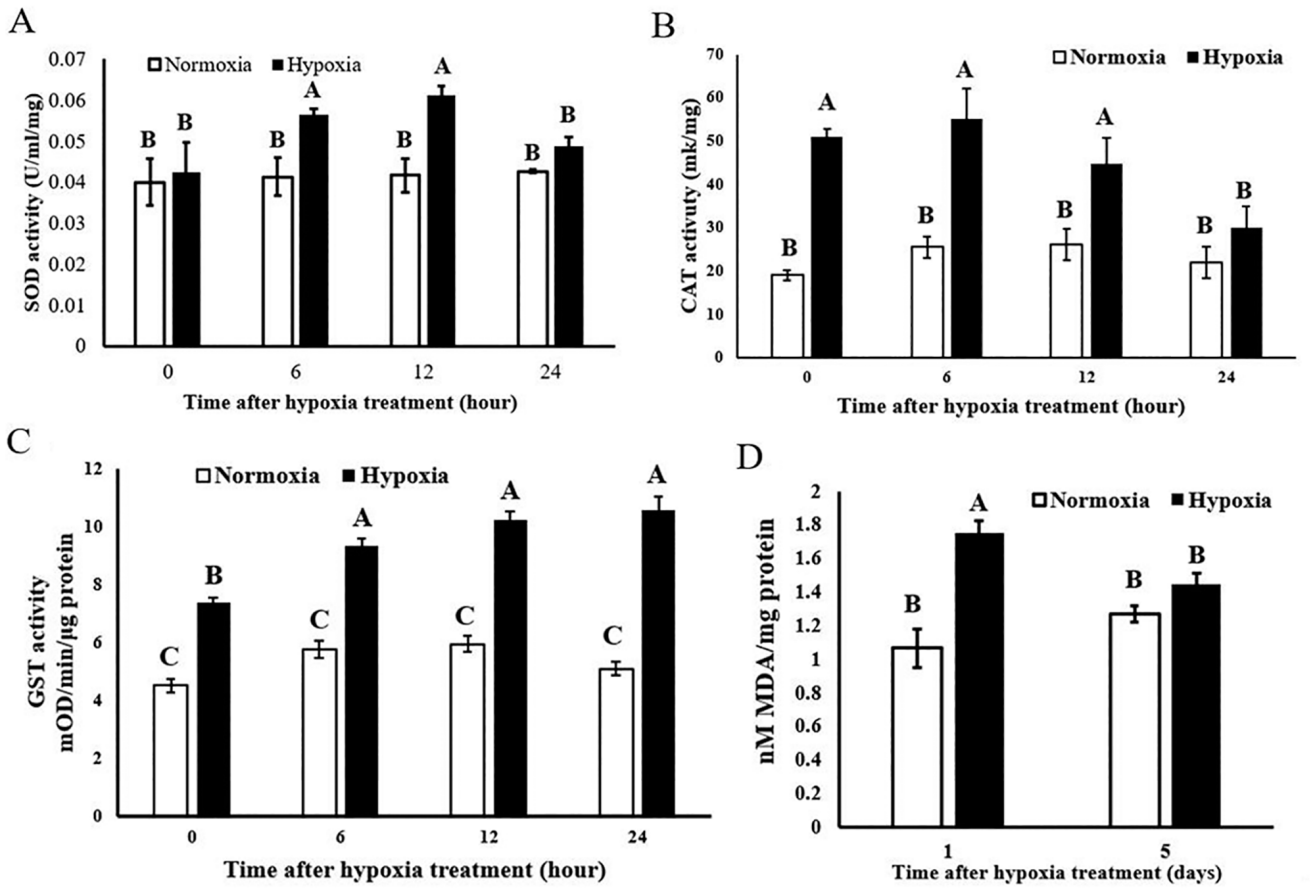
The activity of (A) SOD, (B) CAT, (C) GST and (D) MDA concentration of *T*. *castaneum* under normoxia and hypoxia-reperfusion. The bars represent means ± SE of three replicates. One-way ANOVA followed by Tukey’s range test was used for pairwise comparison of the difference between groups for mean separation (*P* < 0.05).

In this study, we determined antioxidant activity to assess the impact of reoxygenation to the larvae. We found an increase in the total antioxidant enzymes capacity after hypoxic larvae were exposed to normoxia. SOD, CAT, and GST activities increased at the beginning and were maintained at a high level, according to our results (Fig 6). In particular, CAT and GST were induced immediately in insects in response to hypoxia-reperfusion. This lasted more than 24 h to tolerate oxidative stress. SOD showed no change when hypoxic insects were exposed to normoxia at 0 h, although it increased at 6 h and lasted for 24 h. These results led us to predict that antioxidant enzymes were induced to defend against the high level of ROS caused by hypoxia-reperfusion. SOD converts superoxide radicals into H_2_O_2_ and molecular oxygen. CAT converts H_2_O_2_ into H_2_O, whereas GST functions as an antioxidant enzyme and removes lipid peroxidation or hydroperoxide products from cells [75, 76]. SOD represents the first line of defense against ROS in both mitochondria and cytosol. The increased CAT and GST activity after hypoxia-reperfusion correlated with the production of H_2_O_2_ resulting from SOD. These antioxidants were induced in response to hypoxia-reperfusion, which suggests that the larvae boosted their antioxidant systems to defend against the high level of ROS. In addition, the MDA concentration was used to quantify lipid hydroperoxide. The results showed that the MDA concentration was higher in the reoxygenated group than the control group on day 1 (Fig 6), although it returned to normal after 5 d. These results indicate that the high level of lipid peroxidation after reoxygenation and the oxidative damage in insects can be repaired within 5 d. Hypoxia-reperfusion can cause severe damage, and the larvae in this study had very efficient antioxidants, including SOD, CAT, and GST, to defend against the oxidative stress that must occur with the reintroduction of oxygen.

Emerging evidences indicated that insects under hypoxia might have prepared antioxidant defenses for dealing with subsequent reoxygenation. ROS increase temporarily at an early phase of hypoxia, which are the signaling molecules that involved in the preparation for subsequent oxidative stress, i.e. reperfusion [77]. This is so called “preparation for oxidative stress” effect of hypoxia stress [78]. ROS are over-produced under hypoxia and induce some changes leading to hypoxic biochemical responses, which might occur by activation of specific transcription factors (i.e. Nrf2, NF-κB, FoxO) and post translation regulation mechanisms. Both of them are prepared enhanced antioxidant defenses for subsequent reperfusion. Besides, the hormetic effect might partly explain the enhanced antioxidant enzymes under hypoxia-reperfusion. In the term of “hormesis” process, organisms that are exposed to sub-lethal stresses can induce enhanced physiological changes in later life, such as higher stress resistance and greater lifespan [79, 80]. The stress hardening, i.e. exposure a brief hypoxia to larvae would lead to enhanced resistance to more severe applications of oxidative resistance in our study.

## Conclusions

Oxygen is essential to aerobic life, and hypoxia can have severe consequences for organisms. Insects have evolved resistance mechanisms to deal with low oxygen. Next-generation sequencing allowed us to investigate these responses at the genetic level. Here we presented the hypoxia transcriptome profiles of *T*. *castaneum* larvae and identified key genes involved in several signal transduction pathways. We used qRT-PCR to measure the transcriptional activities of genes involved in glycolysis and the Krebs cycle. Our results showed that the beetles could shift to anaerobic metabolism in response to hypoxia. The results also showed that hypoxia suppressed citrate synthase and might have led to mitochondrial autophagy in insects, yet the larvae recovered from hypoxic stress within a short time. Hypoxia-reperfusion induced high oxidative stress in insects, and the activities of antioxidative enzymes such as SOD, CAT, and GST increased to tolerate oxidative stress. Together our findings promote a better understanding of how low oxygen affects signal transduction in insects. In addition, our research makes it possible to develop more efficient strategies to control storage insects. To achieve high confidence of pest control, other environment factors that may affect modified atmosphere efficacy, such as temperature, humidity, irradiation should be taken into consideration.

## Supporting information

S1 Fig The “volcano plot” picture of differentially expressed genes. (DOC)

S2 Fig The KEGG pathway analysis of genes involves in the hypoxia responses. (DOC)

S1 Table Summary of the RNA-seq raw data in *Tribolium castaneum*. (DOCX)

S2 Table Differentially expressed genes indicated by the FPKM statistical analysis. (DOCX)
