# Supplementary material for: Inhibition of mitochondrial respiration under hypoxia and increased antioxidant activity after reoxygenation of Tribolium castaneum
Source: PLoS One. 2018 Jun 14;13(6):e0199056. doi: 10.1371/journal.pone.0199056 (PMC6002095; doi:10.1371/journal.pone.0199056)
Supplement: S1 Fig — (DOC) [file pone.0199056.s001.doc]

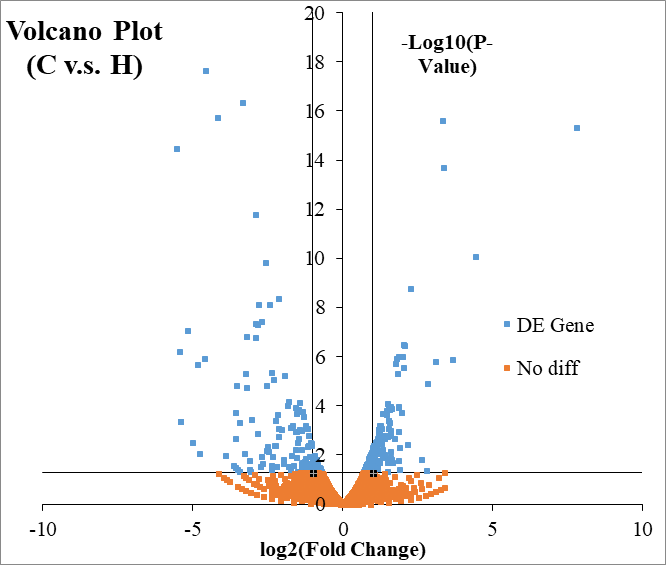


S1 Fig. The “volcano plot” picture of differentially expressed genes from two groups. TC1, the control group; TC2, the hypoxia treated group.
