# Supplementary figures and images for: Inhibition of mitochondrial respiration under hypoxia and increased antioxidant activity after reoxygenation of Tribolium castaneum
Source: PLoS One. 2018 Jun 14;13(6):e0199056. doi: 10.1371/journal.pone.0199056 (PMC6002095; doi:10.1371/journal.pone.0199056)

**
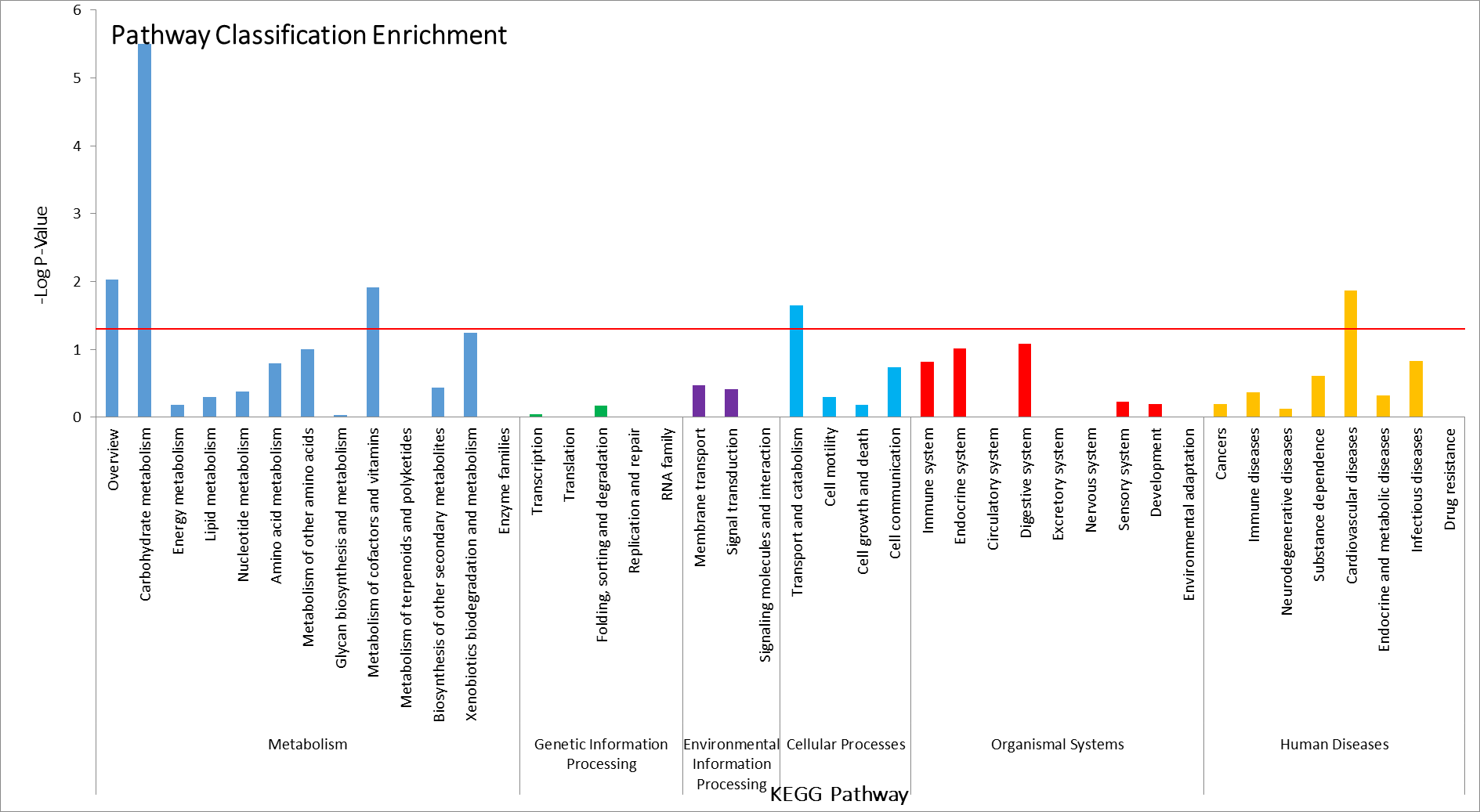
**

S2 Fig. The KEGG pathway analysis of genes involves in the hypoxia responses.

Supplement: S2 Fig — (DOC) [file pone.0199056.s002.doc]
