# Supplementary material for: Inhibition of mitochondrial respiration under hypoxia and increased antioxidant activity after reoxygenation of Tribolium castaneum
Source: PLoS One. 2018 Jun 14;13(6):e0199056. doi: 10.1371/journal.pone.0199056 (PMC6002095; doi:10.1371/journal.pone.0199056)
Supplement: S1 Table — (DOCX) [file pone.0199056.s003.docx]

|  |  |  |  |  |
| --- | --- | --- | --- | --- |
| **S1 Table. Summary of the RNA-Seq raw data in *Tribolium castaneum*** | | | |  |
|  |  |  |  |  |
| Sample | Raw Reads | Clean Reads | Useful Reads % | GC % |
| Control | 17,307,627 | 12,524,554 | 72.36% | 48.09% |
| Hypoxia/hypercapnia | 19,866,284 | 14,193,339 | 71.44% | 50.74% |
|  |  |  |  |  |
|  |  |  |  |  |
