# Supplementary material for: Inhibition of mitochondrial respiration under hypoxia and increased antioxidant activity after reoxygenation of Tribolium castaneum
Source: PLoS One. 2018 Jun 14;13(6):e0199056. doi: 10.1371/journal.pone.0199056 (PMC6002095; doi:10.1371/journal.pone.0199056)
Supplement: S2 Table — (DOCX) [file pone.0199056.s004.docx]

| **S2 Table. Differentially expressed genes indicated by the FPKM statistical analysis.** | | | | |
| --- | --- | --- | --- | --- |
|  |  |  |  |  |
| **Gene ID** | **log2FoldChange (Hypoxia/Normoxia)** | ***p*-val** | **FDR** | **Annotation** |
| TC000326 | -1.08 | 3.16E-03 | 1.43E-05 | Putative uncharacterized protein[Source:UniProtKB/TrEMBL;Acc:D6WAW8] |
| TC000544 | -0.92 | 4.55E-02 | 2.61E-38 | Putative uncharacterized protein[Source:UniProtKB/TrEMBL;Acc:D6W9P2] |
| TC000547 | -1.57 | 1.09E-03 | 4.90E-27 | Serine protease P13[Source:UniProtKB/TrEMBL;Acc:D6W9N8] |
| TC000639 | -2.38 | 4.25E-06 | 1.90E-29 | Putative uncharacterized protein[Source:UniProtKB/TrEMBL;Acc:D6W958] |
| TC000829 | -1.00 | 2.66E-02 | 7.00E-05 | Serine protease H18[Source:UniProtKB/TrEMBL;Acc:D6W8K4] |
| TC000865 | -2.20 | 3.01E-02 | 1.12E-55 | Putative uncharacterized protein[Source:UniProtKB/TrEMBL;Acc:D6W8U3] |
| TC000936 | -1.46 | 1.42E-04 | 1.64E-08 | Putative uncharacterized protein[Source:UniProtKB/TrEMBL;Acc:D6W959] |
| TC000937 | -1.19 | 8.53E-04 | 6.48E-141 | Alpha-amylase [Source:UniProtKB/Swiss-Prot;Acc:P09107] |
| TC000938 | -1.31 | 2.85E-04 | 9.56E-04 | Putative uncharacterized protein[Source:UniProtKB/TrEMBL;Acc:D6W961] |
| TC000939 | -1.13 | 1.65E-03 | 9.55E-04 | Putative uncharacterized protein[Source:UniProtKB/TrEMBL;Acc:D6W962] |
| TC000940 | -1.05 | 4.01E-03 | 1.04E-23 | Putative uncharacterized protein[Source:UniProtKB/TrEMBL;Acc:D6W963] |
| TC000944 | -1.37 | 2.76E-02 | 1.15E-142 | Putative uncharacterized protein[Source:UniProtKB/TrEMBL;Acc:D6W968] |
| TC001038 | -1.22 | 2.79E-02 | 1.27E-13 | Putative uncharacterized protein[Source:UniProtKB/TrEMBL;Acc:D6W9R3] |
| TC001175 | -2.71 | 3.83E-08 | 0.00E+00 | Putative uncharacterized protein[Source:UniProtKB/TrEMBL;Acc:D6WAL5] |
| TC001350 | -1.57 | 1.17E-04 | 2.27E-19 | Obstractor D [Source:UniProtKB/TrEMBL;Acc:A1YR30]" |
| TC001352 | -0.99 | 1.58E-02 | 6.74E-04 | Putative uncharacterized protein[Source:UniProtKB/TrEMBL;Acc:D6WCB4] |
| TC001770 | -5.55 | 3.51E-15 | 9.15E-28 | Putative uncharacterized protein[Source:UniProtKB/TrEMBL;Acc:D6W8E2] |
| TC001793 | -1.54 | 1.24E-02 | 9.59E-08 | Putative uncharacterized protein[Source:UniProtKB/TrEMBL;Acc:D7EKV0] |
| TC001910 | -3.06 | 4.65E-02 | 3.21E-130 | Putative uncharacterized protein[Source:UniProtKB/TrEMBL;Acc:D7GYM6] |
| TC002389 | -0.84 | 3.82E-02 | 1.17E-05 | Putative uncharacterized protein[Source:UniProtKB/TrEMBL;Acc:D6WIX3] |
| TC002620 | -2.38 | 3.99E-02 | 3.26E-18 | Putative uncharacterized protein[Source:UniProtKB/TrEMBL;Acc:D6WF57] |
| TC002621 | -3.45 | 4.73E-04 | 1.55E-73 | Putative uncharacterized protein[Source:UniProtKB/TrEMBL;Acc:D6WF56] |
| TC002761 | -3.59 | 1.95E-04 | 2.36E-05 | Putative uncharacterized protein[Source:UniProtKB/TrEMBL;Acc:D6WDJ9] |
| TC002785 | -2.45 | 7.53E-09 | 4.12E-52 | Serine protease P40[Source:UniProtKB/TrEMBL;Acc:D6WIP3] |
| TC002952 | -1.05 | 3.40E-03 | 5.72E-05 | Cathepsin B [Source:UniProtKB/TrEMBL;Acc:D6WGZ4]" |
| TC003047 | -4.59 | 2.34E-18 | 1.94E-06 | Putative uncharacterized protein[Source:UniProtKB/TrEMBL;Acc:D6WG24] |
| TC003050 | -1.01 | 4.86E-02 | 6.17E-43 | Putative uncharacterized protein[Source:UniProtKB/TrEMBL;Acc:D6WG19] |
| TC003060 | -1.15 | 4.44E-02 | 2.62E-09 | Putative uncharacterized protein[Source:UniProtKB/TrEMBL;Acc:D6WFU1] |
| TC003391 | -0.88 | 1.90E-02 | 4.64E-07 | Putative uncharacterized protein[Source:UniProtKB/TrEMBL;Acc:D6WG44] |
| TC003944 | -2.58 | 1.53E-10 | 3.59E-60 | Putative uncharacterized protein[Source:UniProtKB/TrEMBL;Acc:D6WHZ9] |
| TC003981 | -2.55 | 6.87E-03 | 2.31E-04 | Putative uncharacterized protein[Source:UniProtKB/TrEMBL;Acc:D6WID9] |
| TC003990 | -1.16 | 3.08E-02 | 1.87E-21 | Putative uncharacterized protein[Source:UniProtKB/TrEMBL;Acc:D6WIF6] |
| TC004000 | -2.86 | 5.01E-08 | 2.30E-05 | Putative uncharacterized protein[Source:UniProtKB/TrEMBL;Acc:D6WIH3] |
| TC004289 | -0.98 | 2.23E-02 | 2.94E-171 | Putative uncharacterized protein[Source:UniProtKB/TrEMBL;Acc:D7EL55] |
| TC004416 | -1.82 | 2.95E-02 | 6.31E-08 | Putative uncharacterized protein[Source:UniProtKB/TrEMBL;Acc:D6WD26] |
| TC004635 | -2.25 | 4.10E-04 | 5.56E-05 | Serine protease P53[Source:UniProtKB/TrEMBL;Acc:D6W7D1] |
| TC004759 | -1.36 | 2.31E-02 | 3.81E-06 | Putative uncharacterized protein[Source:UniProtKB/TrEMBL;Acc:D6W7Y4] |
| TC004842 | -0.86 | 1.53E-02 | 1.08E-62 | Putative uncharacterized protein[Source:UniProtKB/TrEMBL;Acc:D6WB12] |
| TC004895 | -1.34 | 4.76E-02 | 0.00E+00 | Toll-6 [Source:UniProtKB/TrEMBL;Acc:D6WCH0]" |
| TC004898 | -1.24 | 2.17E-02 | 0.00E+00 | Tollo [Source:UniProtKB/TrEMBL;Acc:D6WCH6]" |
| TC005181 | -1.54 | 1.97E-04 | 3.12E-23 | Putative uncharacterized protein[Source:UniProtKB/TrEMBL;Acc:D6W7E9] |
| TC005190 | -0.93 | 1.39E-02 | 2.67E-04 | Esterase 6 [Source:UniProtKB/TrEMBL;Acc:D7ELD3]" |
| TC005377 | -1.44 | 7.22E-05 | 9.03E-04 | Putative uncharacterized protein[Source:UniProtKB/TrEMBL;Acc:D6WUQ7] |
| TC005740 | -0.79 | 4.74E-02 | 1.83E-06 | Serpin peptidase inhibitor 7[Source:UniProtKB/TrEMBL;Acc:D6WWI0] |
| TC005941 | -2.36 | 1.20E-02 | 4.35E-07 | Putative uncharacterized protein[Source:UniProtKB/TrEMBL;Acc:D6WVF9] |
| TC006027 | -2.81 | 7.43E-09 | 3.18E-11 | Putative uncharacterized protein[Source:UniProtKB/TrEMBL;Acc:D6WUI2] |
| TC006107 | -0.86 | 2.48E-02 | 1.85E-16 | Putative uncharacterized protein[Source:UniProtKB/TrEMBL;Acc:D6WYT0] |
| TC006224 | -1.98 | 1.49E-02 | 1.15E-60 | Putative uncharacterized protein[Source:UniProtKB/TrEMBL;Acc:D6WVH8] |
| TC006254 | -0.94 | 1.02E-02 | 6.34E-04 | Putative uncharacterized protein[Source:UniProtKB/TrEMBL;Acc:D6WVP0] |
| TC006316 | -3.21 | 1.57E-07 | 3.05E-06 | Putative uncharacterized protein[Source:UniProtKB/TrEMBL;Acc:D6WW23] |
| TC006317 | -2.74 | 3.05E-02 | 1.29E-04 | Putative uncharacterized protein[Source:UniProtKB/TrEMBL;Acc:D6WW24] |
| TC006543 | -4.76 | 8.73E-03 | 4.43E-04 | Pyruvate kinase[Source:UniProtKB/TrEMBL;Acc:D6WXH3] |
| TC006703 | -3.12 | 3.93E-02 | 1.03E-200 | Putative uncharacterized protein[Source:UniProtKB/TrEMBL;Acc:D6WYJ3] |
| TC006749 | -2.03 | 9.05E-04 | 1.50E-175 | Putative uncharacterized protein[Source:UniProtKB/TrEMBL;Acc:D6WZ13] |
| TC006777 | -1.00 | 4.12E-02 | 0.00E+00 | Putative uncharacterized protein[Source:UniProtKB/TrEMBL;Acc:D6WUS8] |
| TC006780 | -1.21 | 2.44E-02 | 6.28E-10 | Putative uncharacterized protein[Source:UniProtKB/TrEMBL;Acc:D6WUU2] |
| TC006978 | -2.31 | 8.16E-06 | 9.88E-63 | Putative uncharacterized protein[Source:UniProtKB/TrEMBL;Acc:D7EJZ5] |
| TC007056 | -1.52 | 1.46E-04 | 9.65E-28 | Putative uncharacterized protein GLEAN_07056[Source:UniProtKB/TrEMBL;Acc:D2A215] |
| TC007179 | -1.30 | 4.72E-02 | 3.42E-142 | Putative uncharacterized protein GLEAN_07179[Source:UniProtKB/TrEMBL;Acc:D2A0X8] |
| TC007207 | -5.44 | 6.42E-07 | 1.16E-06 | Putative uncharacterized protein GLEAN_07207[Source:UniProtKB/TrEMBL;Acc:D2A0T4] |
| TC007265 | -2.13 | 1.84E-03 | 1.46E-13 | Putative uncharacterized protein GLEAN_07265[Source:UniProtKB/TrEMBL;Acc:D2A0G8] |
| TC007273 | -5.42 | 4.15E-04 | 9.64E-07 | Putative uncharacterized protein GLEAN_07273[Source:UniProtKB/TrEMBL;Acc:D2A0F6] |
| TC007275 | -2.85 | 1.33E-03 | 5.08E-09 | Putative uncharacterized protein GLEAN_07275[Source:UniProtKB/TrEMBL;Acc:D2A0F4] |
| TC007326 | -2.55 | 1.44E-05 | 4.23E-09 | Putative uncharacterized protein GLEAN_07326[Source:UniProtKB/TrEMBL;Acc:D2A085] |
| TC007453 | -1.10 | 2.74E-02 | 6.08E-16 | Putative uncharacterized protein GLEAN_07453[Source:UniProtKB/TrEMBL;Acc:D1ZZH5] |
| TC007539 | -1.39 | 5.87E-03 | 3.82E-07 | Alkaline phosphatase[Source:UniProtKB/TrEMBL;Acc:D2A3C2] |
| TC007642 | -1.34 | 2.02E-02 | 5.90E-05 | Putative uncharacterized protein GLEAN_07642[Source:UniProtKB/TrEMBL;Acc:D2A2I8] |
| TC007916 | -1.68 | 6.48E-04 | 3.62E-25 | Putative uncharacterized protein GLEAN_07916[Source:UniProtKB/TrEMBL;Acc:D2A312] |
| TC007979 | -5.42 | 4.15E-04 | 2.93E-04 | Putative uncharacterized protein GLEAN_07979[Source:UniProtKB/TrEMBL;Acc:D2A3G4] |
| TC008268 | -3.21 | 1.78E-05 | 1.39E-91 | Putative uncharacterized protein GLEAN_08268[Source:UniProtKB/TrEMBL;Acc:D2A0U1] |
| TC008357 | -1.49 | 3.17E-03 | 2.13E-33 | Putative uncharacterized protein GLEAN_08357[Source:UniProtKB/TrEMBL;Acc:D2A1A2] |
| TC008800 | -0.97 | 3.66E-02 | 2.45E-04 | Putative uncharacterized protein[Source:UniProtKB/TrEMBL;Acc:D6WRL5] |
| TC008952 | -1.51 | 4.65E-02 | 1.82E-32 | Engrailed [Source:UniProtKB/TrEMBL;Acc:D6WQ96]" |
| TC008954 | -1.14 | 4.19E-03 | 1.84E-197 | Putative uncharacterized protein[Source:UniProtKB/TrEMBL;Acc:D6WQ92] |
| TC009035 | -2.18 | 2.27E-04 | 2.34E-05 | Putative uncharacterized protein[Source:UniProtKB/TrEMBL;Acc:D6WPR3] |
| TC009176 | -0.72 | 4.75E-02 | 1.97E-18 | Chitinase 16 [Source:UniProtKB/TrEMBL;Acc:Q5FYY7]" |
| TC009177 | -1.17 | 4.33E-03 | 8.26E-04 | Chitinase 9 [Source:UniProtKB/TrEMBL;Acc:Q0Z940]" |
| TC009199 | -3.54 | 1.52E-05 | 8.26E-04 | Putative uncharacterized protein[Source:UniProtKB/TrEMBL;Acc:D6WSZ6] |
| TC009350 | -1.14 | 3.18E-02 | 2.93E-11 | Putative uncharacterized protein[Source:UniProtKB/TrEMBL;Acc:D6WRE1] |
| TC009362 | -1.10 | 1.11E-02 | 5.91E-08 | Cathepsin L [Source:UniProtKB/TrEMBL;Acc:D6WR27]" |
| TC009448 | -3.45 | 4.75E-02 | 1.35E-05 | Cathepsin L [Source:UniProtKB/TrEMBL;Acc:D6WRB5]" |
| TC009456 | -0.85 | 4.02E-02 | 5.77E-04 | Putative uncharacterized protein[Source:UniProtKB/TrEMBL;Acc:D6WRC6] |
| TC009633 | -0.75 | 4.57E-02 | 5.53E-26 | Putative uncharacterized protein[Source:UniProtKB/TrEMBL;Acc:D6WTD7] |
| TC009681 | -1.30 | 4.20E-02 | 8.73E-07 | Putative uncharacterized protein[Source:UniProtKB/TrEMBL;Acc:D6WU23] |
| TC009692 | -2.26 | 4.26E-03 | 9.81E-08 | Putative uncharacterized protein[Source:UniProtKB/TrEMBL;Acc:D6WU40] |
| TC009703 | -0.91 | 2.14E-02 | 4.45E-48 | Putative uncharacterized protein[Source:UniProtKB/TrEMBL;Acc:D6WU51] |
| TC009724 | -1.36 | 9.78E-04 | 7.25E-07 | Putative uncharacterized protein[Source:UniProtKB/TrEMBL;Acc:D6WU98] |
| TC009833 | -0.88 | 3.44E-02 | 5.31E-06 | Putative uncharacterized protein[Source:UniProtKB/TrEMBL;Acc:D6WPX1] |
| TC009861 | -2.13 | 8.02E-04 | 8.08E-22 | Putative uncharacterized protein[Source:UniProtKB/TrEMBL;Acc:D6WQ31] |
| TC009896 | -1.10 | 4.33E-02 | 6.75E-08 | Putative uncharacterized protein[Source:UniProtKB/TrEMBL;Acc:D6WQ94] |
| TC009948 | -2.71 | 1.16E-02 | 6.23E-38 | Putative uncharacterized protein[Source:UniProtKB/TrEMBL;Acc:D6WQL1] |
| TC009996 | -0.94 | 4.90E-02 | 4.90E-41 | Putative uncharacterized protein[Source:UniProtKB/TrEMBL;Acc:D6WR61] |
| TC010023 | -2.50 | 4.37E-03 | 4.29E-12 | Putative uncharacterized protein[Source:UniProtKB/TrEMBL;Acc:D6WRK4] |
| TC010052 | -1.36 | 1.75E-04 | 1.74E-06 | Putative uncharacterized protein[Source:UniProtKB/TrEMBL;Acc:D6WRS6] |
| TC010063 | -1.46 | 2.21E-03 | 1.33E-04 | Odorant binding protein 10[Source:UniProtKB/TrEMBL;Acc:D6WS37] |
| TC010066 | -3.33 | 4.66E-17 | 1.27E-05 | Odorant binding protein (Subfamily minus-C) C01[Source:UniProtKB/TrEMBL;Acc:D6WS42] |
| TC010117 | -1.09 | 2.24E-02 | 1.08E-192 | Putative uncharacterized protein[Source:UniProtKB/TrEMBL;Acc:D6WSM1] |
| TC010260 | -2.46 | 7.40E-03 | 0.00E+00 | Putative uncharacterized protein[Source:UniProtKB/TrEMBL;Acc:D7EJS3] |
| TC010304 | -1.72 | 1.08E-02 | 0.00E+00 | Putative uncharacterized protein[Source:UniProtKB/TrEMBL;Acc:D7GY59] |
| TC010419 | -2.23 | 4.03E-03 | 5.27E-18 | Putative uncharacterized protein[Source:UniProtKB/TrEMBL;Acc:D6WKP2] |
| TC010421 | -3.59 | 2.06E-03 | 5.46E-04 | Putative uncharacterized protein[Source:UniProtKB/TrEMBL;Acc:D6WKN9] |
| TC010547 | -1.53 | 1.16E-03 | 5.46E-04 | Putative uncharacterized protein[Source:UniProtKB/TrEMBL;Acc:D6WE16] |
| TC010557 | -0.86 | 2.66E-02 | 4.56E-04 | Putative uncharacterized protein[Source:UniProtKB/TrEMBL;Acc:D6WE66] |
| TC011140 | -1.84 | 9.95E-05 | 3.03E-05 | Cuticular protein analogous to peritrophins 3-A1[Source:UniProtKB/TrEMBL;Acc:D1MAI4] |
| TC011522 | -0.75 | 3.49E-02 | 4.69E-09 | Putative uncharacterized protein[Source:UniProtKB/TrEMBL;Acc:D6W6D2] |
| TC012047 | -3.92 | 1.08E-02 | 1.61E-11 | Putative uncharacterized protein[Source:UniProtKB/TrEMBL;Acc:D6X281] |
| TC012049 | -3.65 | 2.74E-02 | 1.54E-31 | Putative uncharacterized protein[Source:UniProtKB/TrEMBL;Acc:D6X279] |
| TC012050 | -3.11 | 1.70E-02 | 6.03E-08 | Putative uncharacterized protein[Source:UniProtKB/TrEMBL;Acc:D6X278] |
| TC012051 | -3.52 | 3.95E-02 | 2.19E-04 | Putative uncharacterized protein[Source:UniProtKB/TrEMBL;Acc:D6X277] |
| TC012154 | -0.89 | 4.31E-02 | 6.69E-65 | Putative uncharacterized protein[Source:UniProtKB/TrEMBL;Acc:D6X103] |
| TC012207 | -6.92 | 4.90E-52 | 1.12E-08 | Putative uncharacterized protein[Source:UniProtKB/TrEMBL;Acc:D6X073] |
| TC012229 | -1.36 | 3.95E-02 | 1.57E-15 | Putative uncharacterized protein[Source:UniProtKB/TrEMBL;Acc:D6X032] |
| TC012337 | -5.02 | 3.14E-03 | 2.98E-20 | Putative uncharacterized protein[Source:UniProtKB/TrEMBL;Acc:D6X1R3] |
| TC012521 | -1.02 | 1.34E-02 | 8.71E-05 | Starry night [Source:UniProtKB/TrEMBL;Acc:D6X315]" |
| TC012546 | -3.23 | 4.65E-06 | 7.23E-05 | Putative uncharacterized protein[Source:UniProtKB/TrEMBL;Acc:D6X353] |
| TC012547 | -4.60 | 1.14E-06 | 7.33E-07 | Putative uncharacterized protein[Source:UniProtKB/TrEMBL;Acc:D6X354] |
| TC012734 | -2.90 | 1.59E-12 | 2.85E-05 | Putative uncharacterized protein[Source:UniProtKB/TrEMBL;Acc:D6WZW3] |
| TC013306 | -4.85 | 2.05E-06 | 8.17E-12 | Putative uncharacterized protein[Source:UniProtKB/TrEMBL;Acc:D6WMA9] |
| TC013418 | -1.80 | 6.64E-05 | 0.00E+00 | Putative uncharacterized protein[Source:UniProtKB/TrEMBL;Acc:D6WLM3] |
| TC013448 | -0.91 | 4.32E-02 | 7.38E-04 | Putative uncharacterized protein[Source:UniProtKB/TrEMBL;Acc:D6WLG3] |
| TC013683 | -0.77 | 3.57E-02 | 6.16E-04 | Putative uncharacterized protein[Source:UniProtKB/TrEMBL;Acc:D6WKD1] |
| TC013741 | -2.15 | 4.16E-09 | 4.34E-56 | Putative uncharacterized protein[Source:UniProtKB/TrEMBL;Acc:D6WJX6] |
| TC014184 | -4.17 | 1.82E-16 | 3.25E-68 | Putative uncharacterized protein[Source:UniProtKB/TrEMBL;Acc:D6W6W7] |
| TC014229 | -1.27 | 1.80E-02 | 9.68E-21 | Putative uncharacterized protein[Source:UniProtKB/TrEMBL;Acc:D6W763] |
| TC014345 | -1.40 | 1.77E-04 | 6.42E-08 | Putative uncharacterized protein[Source:UniProtKB/TrEMBL;Acc:D6WLG2] |
| TC014588 | -1.76 | 4.36E-02 | 3.06E-06 | Citrate synthase[Source:UniProtKB/TrEMBL;Acc:D6WMM6] |
| TC014609 | -0.78 | 3.29E-02 | 2.53E-06 | Putative uncharacterized protein[Source:UniProtKB/TrEMBL;Acc:D6WMX6] |
| TC014768 | -2.69 | 2.15E-02 | 2.69E-32 | Putative uncharacterized protein[Source:UniProtKB/TrEMBL;Acc:D6WJM4] |
| TC014944 | -3.27 | 8.93E-03 | 9.84E-05 | Putative uncharacterized protein GLEAN_14944[Source:UniProtKB/TrEMBL;Acc:D2A3U7] |
| TC014970 | -2.38 | 2.80E-02 | 1.03E-13 | Putative uncharacterized protein GLEAN_14970[Source:UniProtKB/TrEMBL;Acc:D2A3M6] |
| TC015132 | -0.94 | 2.27E-02 | 5.64E-05 | Putative uncharacterized protein GLEAN_15132[Source:UniProtKB/TrEMBL;Acc:D2A5Q3] |
| TC015245 | -2.89 | 4.29E-08 | 3.37E-26 | Putative uncharacterized protein GLEAN_15245[Source:UniProtKB/TrEMBL;Acc:D2A557] |
| TC015312 | -1.36 | 2.73E-02 | 2.22E-05 | Putative uncharacterized protein GLEAN_15312[Source:UniProtKB/TrEMBL;Acc:D2A4T1] |
| TC015340 | -0.94 | 2.09E-02 | 7.78E-36 | Putative uncharacterized protein GLEAN_15340[Source:UniProtKB/TrEMBL;Acc:D2A4M4] |
| TC015400 | -1.45 | 8.86E-04 | 1.61E-51 | Putative uncharacterized protein GLEAN_15400[Source:UniProtKB/TrEMBL;Acc:D2A4P5] |
| TC015465 | -0.89 | 2.70E-02 | 8.00E-56 | Putative uncharacterized protein GLEAN_15465[Source:UniProtKB/TrEMBL;Acc:D2A533] |
| TC015492 | -1.24 | 3.53E-02 | 7.99E-14 | Putative uncharacterized protein GLEAN_15492[Source:UniProtKB/TrEMBL;Acc:D2A589] |
| TC015587 | -3.04 | 3.72E-04 | 4.78E-22 | Putative uncharacterized protein GLEAN_15587[Source:UniProtKB/TrEMBL;Acc:D2A5S6] |
| TC015589 | -2.91 | 1.65E-07 | 1.35E-08 | Putative uncharacterized protein GLEAN_15589[Source:UniProtKB/TrEMBL;Acc:D2A5S8] |
| TC015601 | -1.24 | 1.36E-02 | 1.27E-17 | Putative uncharacterized protein GLEAN_15601[Source:UniProtKB/TrEMBL;Acc:D2A5V9] |
| TC015612 | -1.24 | 2.87E-02 | 0.00E+00 | Putative uncharacterized protein GLEAN_15612[Source:UniProtKB/TrEMBL;Acc:D2A5X7] |
| TC015614 | -1.25 | 6.56E-04 | 1.92E-29 | Putative uncharacterized protein GLEAN_15614[Source:UniProtKB/TrEMBL;Acc:D2A5X9] |
| TC015670 | -0.86 | 3.10E-02 | 1.68E-33 | Serine protease H164[Source:UniProtKB/TrEMBL;Acc:D2A6A6] |
| TC015722 | -1.93 | 5.99E-06 | 5.83E-68 | Putative uncharacterized protein GLEAN_15722[Source:UniProtKB/TrEMBL;Acc:D2A3N5] |
| TC015723 | -1.14 | 4.17E-03 | 0.00E+00 | Putative uncharacterized protein GLEAN_15723[Source:UniProtKB/TrEMBL;Acc:D2A3N6] |
| TC015805 | -1.57 | 6.24E-03 | 3.34E-04 | Myoglianin [Source:UniProtKB/TrEMBL;Acc:D2A418]" |
| TC015951 | -3.59 | 3.29E-02 | 1.13E-175 | Putative uncharacterized protein[Source:UniProtKB/TrEMBL;Acc:D6WTZ5] |
| TC016058 | -1.63 | 2.60E-02 | 1.33E-04 | Putative uncharacterized protein[Source:UniProtKB/TrEMBL;Acc:D6W6T0] |
| TC016235 | -0.97 | 1.84E-02 | 7.77E-39 | Putative uncharacterized protein[Source:UniProtKB/TrEMBL;Acc:D6X4T2] |
| TC016278 | -1.97 | 2.16E-02 | 9.21E-05 | Putative uncharacterized protein[Source:UniProtKB/TrEMBL;Acc:D6X2U7] |
| TC016313 | -1.74 | 7.98E-04 | 3.63E-05 | Putative uncharacterized protein[Source:UniProtKB/TrEMBL;Acc:D6WMS2] |
| TC016377 | -5.16 | 8.48E-08 | 3.01E-05 | Putative uncharacterized protein[Source:UniProtKB/TrEMBL;Acc:D6WPB7] |
| TC030732 | -2.20 | 3.01E-02 | 3.38E-274 | Putative uncharacterized protein[Source:UniProtKB/TrEMBL;Acc:D6WGY5] |
| TC030784 | -1.17 | 3.65E-02 | 1.15E-32 | Putative uncharacterized protein[Source:UniProtKB/TrEMBL;Acc:D6WE92] |
| TC000087 | 0.80 | 2.56E-02 | 4.37E-08 | Putative uncharacterized protein[Source:UniProtKB/TrEMBL;Acc:D6WIQ7] |
| TC000319 | 0.90 | 1.19E-02 | 5.23E-08 | Putative uncharacterized protein[Source:UniProtKB/TrEMBL;Acc:D6WAX9] |
| TC000403 | 0.95 | 7.95E-03 | 6.59E-04 | Branched-chain-amino-acid aminotransferase[Source:UniProtKB/TrEMBL;Acc:D6WAG1] |
| TC000500 | 1.22 | 1.62E-02 | 6.59E-04 | Antimicrobial peptide[Source:UniProtKB/TrEMBL;Acc:D6W9Y9] |
| TC000542 | 1.22 | 6.40E-04 | 1.15E-04 | Phosphoserine aminotransferase[Source:UniProtKB/TrEMBL;Acc:D6W9Q3] |
| TC000573 | 0.95 | 1.88E-02 | 8.12E-06 | Putative uncharacterized protein[Source:UniProtKB/TrEMBL;Acc:D6W9I9] |
| TC000626 | 0.93 | 2.59E-02 | 5.67E-07 | Putative uncharacterized protein[Source:UniProtKB/TrEMBL;Acc:D6W994] |
| TC000948 | 1.13 | 7.02E-03 | 5.72E-04 | Putative uncharacterized protein[Source:UniProtKB/TrEMBL;Acc:D6W974] |
| TC000982 | 1.33 | 8.31E-03 | 1.70E-05 | Putative uncharacterized protein[Source:UniProtKB/TrEMBL;Acc:D6W9E8] |
| TC001293 | 1.07 | 5.42E-03 | 1.19E-18 | Putative uncharacterized protein[Source:UniProtKB/TrEMBL;Acc:D6WBR5] |
| TC001449 | 0.77 | 3.52E-02 | 1.38E-08 | Semaphorin-5c[Source:UniProtKB/TrEMBL;Acc:D7EII6] |
| TC001577 | 1.07 | 4.41E-03 | 2.40E-04 | Putative uncharacterized protein[Source:UniProtKB/TrEMBL;Acc:D6WFS6] |
| TC001581 | 1.41 | 1.30E-02 | 9.97E-05 | Putative uncharacterized protein[Source:UniProtKB/TrEMBL;Acc:D6WFT9] |
| TC001705 | 1.89 | 5.04E-03 | 4.16E-19 | Branchless [Source:UniProtKB/TrEMBL;Acc:D6W8C7]" |
| TC001760 | 1.74 | 2.07E-03 | 4.58E-14 | Branchless [Source:UniProtKB/TrEMBL;Acc:D6W8C8]" |
| TC001784 | 0.86 | 4.17E-02 | 4.87E-39 | Putative uncharacterized protein[Source:UniProtKB/TrEMBL;Acc:D7GXR3] |
| TC001813 | 0.76 | 3.72E-02 | 2.02E-09 | Putative uncharacterized protein[Source:UniProtKB/TrEMBL;Acc:D7EKJ1] |
| TC002340 | 1.15 | 2.45E-03 | 2.91E-08 | Putative uncharacterized protein[Source:UniProtKB/TrEMBL;Acc:D7EJH1] |
| TC002431 | 1.20 | 9.34E-04 | 6.06E-06 | Putative uncharacterized protein[Source:UniProtKB/TrEMBL;Acc:D6WIG0] |
| TC002496 | 1.19 | 1.09E-03 | 9.65E-16 | Putative uncharacterized protein[Source:UniProtKB/TrEMBL;Acc:D6WHA4] |
| TC002681 | 1.12 | 2.86E-03 | 8.62E-05 | Putative uncharacterized protein[Source:UniProtKB/TrEMBL;Acc:D6WEB0] |
| TC002779 | 0.76 | 3.12E-02 | 1.03E-08 | Putative uncharacterized protein[Source:UniProtKB/TrEMBL;Acc:D6WDF5] |
| TC002911 | 1.46 | 1.53E-04 | 1.03E-08 | Putative uncharacterized protein[Source:UniProtKB/TrEMBL;Acc:D6WHF9] |
| TC002942 | 1.29 | 2.05E-03 | 2.07E-04 | Putative uncharacterized protein[Source:UniProtKB/TrEMBL;Acc:D6WH17] |
| TC002953 | 0.81 | 4.00E-02 | 1.49E-07 | Cathepsin B [Source:UniProtKB/TrEMBL;Acc:D6WGZ3]" |
| TC002954 | 2.01 | 1.01E-06 | 4.96E-04 | Cathepsin B [Source:UniProtKB/TrEMBL;Acc:D6WGZ2]" |
| TC002976 | 0.79 | 3.26E-02 | 2.15E-06 | Putative uncharacterized protein[Source:UniProtKB/TrEMBL;Acc:D6WGN4] |
| TC002990 | 0.91 | 9.88E-03 | 4.81E-23 | Ribose-phosphate pyrophosphokinase[Source:UniProtKB/TrEMBL;Acc:D6WGK0] |
| TC003044 | 0.87 | 1.43E-02 | 8.90E-09 | Putative uncharacterized protein[Source:UniProtKB/TrEMBL;Acc:D6WG27] |
| TC003289 | 1.11 | 6.22E-03 | 2.20E-10 | Putative uncharacterized protein[Source:UniProtKB/TrEMBL;Acc:D6WET7] |
| TC003387 | 1.20 | 9.43E-04 | 3.07E-05 | Putative uncharacterized protein[Source:UniProtKB/TrEMBL;Acc:D6WG32] |
| TC003468 | 0.87 | 2.22E-02 | 3.15E-19 | Putative uncharacterized protein[Source:UniProtKB/TrEMBL;Acc:D6WGW0] |
| TC003541 | 2.80 | 4.30E-02 | 9.97E-36 | Putative uncharacterized protein[Source:UniProtKB/TrEMBL;Acc:D6WHH1] |
| TC003922 | 1.23 | 2.32E-03 | 4.30E-04 | Putative uncharacterized protein[Source:UniProtKB/TrEMBL;Acc:D6WHN0] |
| TC003997 | 1.19 | 1.55E-03 | 1.58E-99 | Putative uncharacterized protein[Source:UniProtKB/TrEMBL;Acc:D6WIG9] |
| TC004059 | 1.39 | 1.00E-02 | 3.33E-128 | Putative uncharacterized protein[Source:UniProtKB/TrEMBL;Acc:D7EIF0] |
| TC004262 | 0.99 | 2.18E-02 | 1.55E-04 | Putative uncharacterized protein GLEAN_04262[Source:UniProtKB/TrEMBL;Acc:D2CG67] |
| TC004436 | 1.32 | 5.93E-03 | 1.59E-26 | Putative uncharacterized protein[Source:UniProtKB/TrEMBL;Acc:D6WCW9] |
| TC004437 | 0.92 | 1.01E-02 | 3.71E-48 | Putative uncharacterized protein[Source:UniProtKB/TrEMBL;Acc:D6WCW8] |
| TC004605 | 0.85 | 2.60E-02 | 8.81E-04 | Putative uncharacterized protein[Source:UniProtKB/TrEMBL;Acc:D6W805] |
| TC004661 | 0.77 | 3.99E-02 | 8.81E-04 | Putative uncharacterized protein[Source:UniProtKB/TrEMBL;Acc:D6W6G2] |
| TC004741 | 0.79 | 4.21E-02 | 1.34E-04 | Putative uncharacterized protein[Source:UniProtKB/TrEMBL;Acc:D6W7V5] |
| TC005093 | 1.20 | 8.77E-03 | 1.76E-19 | Putative uncharacterized protein GLEAN_05093[Source:UniProtKB/TrEMBL;Acc:D2CFY5] |
| TC005096 | 1.60 | 9.99E-04 | 2.90E-06 | Putative uncharacterized protein GLEAN_05096[Source:UniProtKB/TrEMBL;Acc:D2CFY8] |
| TC005222 | 1.16 | 3.69E-02 | 7.51E-66 | transcript_id=TC005222-RA" |
| TC005358 | 1.76 | 1.90E-06 | 1.26E-29 | Putative uncharacterized protein[Source:UniProtKB/TrEMBL;Acc:D6WUT7] |
| TC005370 | 1.21 | 8.83E-04 | 7.07E-06 | Putative uncharacterized protein[Source:UniProtKB/TrEMBL;Acc:D6WUR7] |
| TC005374 | 0.73 | 4.29E-02 | 3.42E-22 | Putative uncharacterized protein[Source:UniProtKB/TrEMBL;Acc:D6WUR1] |
| TC005384 | 1.86 | 1.72E-02 | 7.64E-04 | Cytochrome P450 9AB1[Source:UniProtKB/TrEMBL;Acc:D6WUP6] |
| TC005436 | 1.52 | 3.86E-04 | 2.78E-04 | Putative uncharacterized protein[Source:UniProtKB/TrEMBL;Acc:D6WYK7] |
| TC005482 | 0.89 | 1.42E-02 | 1.14E-15 | Putative uncharacterized protein[Source:UniProtKB/TrEMBL;Acc:D6WY20] |
| TC005483 | 1.05 | 3.94E-03 | 1.91E-20 | Putative uncharacterized protein[Source:UniProtKB/TrEMBL;Acc:D6WY19] |
| TC005632 | 1.05 | 1.40E-02 | 1.00E-04 | Putative uncharacterized protein[Source:UniProtKB/TrEMBL;Acc:D6WX56] |
| TC005767 | 1.02 | 5.37E-03 | 0.00E+00 | Putative uncharacterized protein[Source:UniProtKB/TrEMBL;Acc:D6WWC9] |
| TC006124 | 0.94 | 3.42E-02 | 7.26E-10 | Putative uncharacterized protein[Source:UniProtKB/TrEMBL;Acc:D6WUH3] |
| TC006157 | 0.75 | 4.97E-02 | 4.52E-39 | Putative uncharacterized protein[Source:UniProtKB/TrEMBL;Acc:D6WUX7] |
| TC006250 | 0.88 | 1.86E-02 | 3.08E-05 | Putative uncharacterized protein[Source:UniProtKB/TrEMBL;Acc:D6WVN3] |
| TC006291 | 1.07 | 4.02E-03 | 2.07E-139 | Putative uncharacterized protein[Source:UniProtKB/TrEMBL;Acc:D6WVX4] |
| TC006358 | 0.85 | 1.73E-02 | 0.00E+00 | Putative uncharacterized protein[Source:UniProtKB/TrEMBL;Acc:D6WWB6] |
| TC006367 | 0.86 | 2.19E-02 | 4.71E-18 | transcript_id=TC006367-RA" |
| TC006417 | 0.99 | 8.32E-03 | 3.24E-13 | Putative uncharacterized protein[Source:UniProtKB/TrEMBL;Acc:D6WWP7] |
| TC006458 | 1.03 | 1.09E-02 | 2.19E-08 | Putative uncharacterized protein[Source:UniProtKB/TrEMBL;Acc:D6WWZ3] |
| TC006459 | 1.21 | 2.28E-03 | 3.38E-06 | Putative uncharacterized protein[Source:UniProtKB/TrEMBL;Acc:D6WWZ4] |
| TC006471 | 0.83 | 2.72E-02 | 1.28E-29 | Putative uncharacterized protein[Source:UniProtKB/TrEMBL;Acc:D6WX25] |
| TC006631 | 1.50 | 8.09E-05 | 4.98E-04 | Putative uncharacterized protein[Source:UniProtKB/TrEMBL;Acc:D6WY02] |
| TC006681 | 1.10 | 3.14E-03 | 1.66E-20 | Putative uncharacterized protein[Source:UniProtKB/TrEMBL;Acc:D6WYF9] |
| TC006713 | 1.09 | 8.34E-03 | 3.17E-27 | Putative uncharacterized protein[Source:UniProtKB/TrEMBL;Acc:D6WYL6] |
| TC006766 | 1.51 | 1.94E-02 | 2.23E-10 | Putative uncharacterized protein[Source:UniProtKB/TrEMBL;Acc:D6WUQ0] |
| TC006910 | 4.42 | 8.61E-11 | 8.92E-07 | Putative uncharacterized protein[Source:UniProtKB/TrEMBL;Acc:D7ELG3] |
| TC006911 | 2.83 | 1.22E-05 | 1.14E-21 | Putative uncharacterized protein[Source:UniProtKB/TrEMBL;Acc:D7ELG2] |
| TC007184 | 0.78 | 3.26E-02 | 1.40E-08 | Putative uncharacterized protein GLEAN_07184[Source:UniProtKB/TrEMBL;Acc:D2A0X4] |
| TC007185 | 1.51 | 8.24E-04 | 1.67E-18 | Lipase [Source:UniProtKB/TrEMBL;Acc:D2A0X3]" |
| TC007470 | 0.75 | 3.67E-02 | 2.73E-07 | Putative uncharacterized protein GLEAN_07470[Source:UniProtKB/TrEMBL;Acc:D1ZZD4] |
| TC007481 | 0.89 | 1.40E-02 | 6.10E-06 | Putative uncharacterized protein GLEAN_07481[Source:UniProtKB/TrEMBL;Acc:D1ZZB2] |
| TC007628 | 0.90 | 1.27E-02 | 4.80E-05 | Putative uncharacterized protein GLEAN_07628[Source:UniProtKB/TrEMBL;Acc:D2A2L1] |
| TC007689 | 1.41 | 2.06E-03 | 4.80E-05 | Putative uncharacterized protein GLEAN_07689[Source:UniProtKB/TrEMBL;Acc:D2A280] |
| TC007737 | 2.04 | 3.32E-07 | 5.28E-92 | Putative uncharacterized protein GLEAN_07737[Source:UniProtKB/TrEMBL;Acc:D2A1H0] |
| TC007738 | 0.75 | 4.01E-02 | 3.72E-04 | Putative uncharacterized protein GLEAN_07738[Source:UniProtKB/TrEMBL;Acc:D2A1G9] |
| TC007764 | 3.09 | 1.57E-06 | 3.72E-04 | Putative uncharacterized protein GLEAN_07764[Source:UniProtKB/TrEMBL;Acc:D2A1R8] |
| TC007848 | 1.01 | 4.88E-03 | 3.72E-04 | Putative uncharacterized protein GLEAN_07848[Source:UniProtKB/TrEMBL;Acc:D2A2F6] |
| TC007958 | 2.27 | 1.65E-09 | 5.95E-43 | Putative uncharacterized protein GLEAN_07958[Source:UniProtKB/TrEMBL;Acc:D2A3C9] |
| TC008057 | 0.89 | 1.36E-02 | 4.99E-25 | Putative uncharacterized protein GLEAN_08057[Source:UniProtKB/TrEMBL;Acc:D1ZZJ7] |
| TC008226 | 7.78 | 4.79E-16 | 1.16E-04 | Putative uncharacterized protein GLEAN_08226[Source:UniProtKB/TrEMBL;Acc:D2A0K9] |
| TC008534 | 1.09 | 3.03E-03 | 1.93E-14 | Putative uncharacterized protein[Source:UniProtKB/TrEMBL;Acc:D7EI87] |
| TC008681 | 1.17 | 2.74E-02 | 0.00E+00 | Chemosensory protein 1; Chemosensory protein 11[Source:UniProtKB/TrEMBL;Acc:Q0MRL2] |
| TC008682 | 1.33 | 2.06E-04 | 0.00E+00 | Chemosensory protein 10; Chemosensory protein 7[Source:UniProtKB/TrEMBL;Acc:Q0MRL3] |
| TC008936 | 0.88 | 1.95E-02 | 8.81E-04 | Putative uncharacterized protein[Source:UniProtKB/TrEMBL;Acc:D6WQB9] |
| TC009099 | 0.75 | 4.18E-02 | 3.57E-05 | Putative uncharacterized protein[Source:UniProtKB/TrEMBL;Acc:D6WP75] |
| TC009114 | 1.53 | 1.08E-04 | 2.78E-04 | Regucalcin [Source:UniProtKB/TrEMBL;Acc:D6WU81]" |
| TC009126 | 1.62 | 1.53E-02 | 1.20E-06 | Heat shock protein 68a; Heat shock protein 68b[Source:UniProtKB/TrEMBL;Acc:D6WU59] |
| TC009127 | 1.00 | 3.07E-02 | 2.78E-04 | Putative uncharacterized protein[Source:UniProtKB/TrEMBL;Acc:D6WU57] |
| TC009132 | 1.26 | 1.66E-03 | 1.40E-08 | HIF prolyl hydroxylase[Source:UniProtKB/TrEMBL;Acc:D6WU52] |
| TC009159 | 0.77 | 4.94E-02 | 7.33E-14 | Putative uncharacterized protein[Source:UniProtKB/TrEMBL;Acc:D6WTQ3] |
| TC009198 | 1.20 | 2.00E-02 | 0.00E+00 | Putative uncharacterized protein[Source:UniProtKB/TrEMBL;Acc:D6WSZ8] |
| TC009220 | 0.98 | 7.57E-03 | 0.00E+00 | Putative uncharacterized protein[Source:UniProtKB/TrEMBL;Acc:D6WSV6] |
| TC009285 | 1.58 | 1.59E-03 | 1.43E-10 | Putative uncharacterized protein[Source:UniProtKB/TrEMBL;Acc:D6WS04] |
| TC009386 | 0.77 | 4.24E-02 | 2.41E-04 | Putative uncharacterized protein[Source:UniProtKB/TrEMBL;Acc:D6WQY5] |
| TC009602 | 0.77 | 3.08E-02 | 2.41E-04 | Serine protease P96[Source:UniProtKB/TrEMBL;Acc:D6WSY4] |
| TC009929 | 1.24 | 1.81E-03 | 1.43E-12 | Putative uncharacterized protein[Source:UniProtKB/TrEMBL;Acc:D6WQG6] |
| TC009956 | 3.67 | 1.25E-06 | 8.91E-07 | ADFb like protein[Source:UniProtKB/TrEMBL;Acc:D6WQM9] |
| TC009957 | 1.46 | 2.33E-04 | 6.79E-21 | Anti-diruetic peptide[Source:UniProtKB/TrEMBL;Acc:D6WQN0] |
| TC010029 | 0.87 | 1.70E-02 | 6.61E-04 | Putative uncharacterized protein[Source:UniProtKB/TrEMBL;Acc:D6WRL3] |
| TC010057 | 1.50 | 1.73E-03 | 8.29E-08 | Putative uncharacterized protein[Source:UniProtKB/TrEMBL;Acc:D6WRT5] |
| TC010172 | 1.11 | 2.36E-02 | 5.73E-04 | Putative uncharacterized protein[Source:UniProtKB/TrEMBL;Acc:D6WT72] |
| TC010353 | 1.13 | 2.80E-02 | 6.44E-145 | Putative uncharacterized protein[Source:UniProtKB/TrEMBL;Acc:D6WGA2] |
| TC010417 | 0.74 | 4.02E-02 | 5.31E-08 | Putative uncharacterized protein[Source:UniProtKB/TrEMBL;Acc:D6WKP6] |
| TC010423 | 1.58 | 1.09E-04 | 0.00E+00 | Cytochrome P450-like protein[Source:UniProtKB/TrEMBL;Acc:D6WKN7] |
| TC010470 | 0.94 | 2.53E-02 | 4.96E-04 | Putative uncharacterized protein[Source:UniProtKB/TrEMBL;Acc:D6WKU2] |
| TC010495 | 1.02 | 4.23E-03 | 1.50E-07 | Putative uncharacterized protein[Source:UniProtKB/TrEMBL;Acc:D6WE67] |
| TC010517 | 1.15 | 2.22E-03 | 9.82E-14 | Putative uncharacterized protein[Source:UniProtKB/TrEMBL;Acc:D6WDY8] |
| TC010766 | 0.80 | 3.32E-02 | 4.25E-07 | Putative uncharacterized protein[Source:UniProtKB/TrEMBL;Acc:D6W7T6] |
| TC010836 | 1.11 | 4.71E-03 | 1.34E-04 | Putative uncharacterized protein[Source:UniProtKB/TrEMBL;Acc:D6W7I2] |
| TC010884 | 0.88 | 1.54E-02 | 1.19E-157 | Putative uncharacterized protein[Source:UniProtKB/TrEMBL;Acc:D6W7T1] |
| TC010885 | 1.43 | 1.68E-03 | 2.73E-09 | Putative uncharacterized protein[Source:UniProtKB/TrEMBL;Acc:D6W7T2] |
| TC010987 | 1.05 | 3.66E-02 | 0.00E+00 | Putative uncharacterized protein[Source:UniProtKB/TrEMBL;Acc:D6X1G0] |
| TC010997 | 1.89 | 3.84E-02 | 2.05E-11 | Putative uncharacterized protein GLEAN_10997[Source:UniProtKB/TrEMBL;Acc:D2CG57] |
| TC011074 | 1.84 | 4.80E-06 | 1.15E-04 | Putative uncharacterized protein[Source:UniProtKB/TrEMBL;Acc:D6X4G6] |
| TC011075 | 1.80 | 1.23E-06 | 8.88E-25 | Putative uncharacterized protein[Source:UniProtKB/TrEMBL;Acc:D6X4G5] |
| TC011616 | 0.95 | 1.22E-02 | 2.57E-16 | Putative uncharacterized protein[Source:UniProtKB/TrEMBL;Acc:D6X1B0] |
| TC011654 | 1.21 | 2.03E-02 | 3.07E-05 | Putative uncharacterized protein[Source:UniProtKB/TrEMBL;Acc:D6X0V1] |
| TC011675 | 1.00 | 7.29E-03 | 3.07E-05 | Superoxide dismutase [Cu-Zn][Source:UniProtKB/TrEMBL;Acc:D6X0S3] |
| TC011730 | 0.95 | 1.93E-02 | 2.76E-04 | Putative uncharacterized protein[Source:UniProtKB/TrEMBL;Acc:D6X017] |
| TC011733 | 1.85 | 1.02E-06 | 2.76E-04 | Putative uncharacterized protein[Source:UniProtKB/TrEMBL;Acc:D6X008] |
| TC011751 | 0.75 | 4.41E-02 | 4.56E-08 | Putative uncharacterized protein[Source:UniProtKB/TrEMBL;Acc:D6WZX8] |
| TC011805 | 0.83 | 2.89E-02 | 2.28E-05 | Putative uncharacterized protein[Source:UniProtKB/TrEMBL;Acc:D6WZN2] |
| TC011937 | 0.83 | 2.60E-02 | 1.90E-12 | Putative uncharacterized protein[Source:UniProtKB/TrEMBL;Acc:D6X366] |
| TC011938 | 1.22 | 1.09E-03 | 1.96E-05 | Putative uncharacterized protein[Source:UniProtKB/TrEMBL;Acc:D6X365] |
| TC012119 | 1.01 | 6.79E-03 | 2.39E-04 | Putative uncharacterized protein[Source:UniProtKB/TrEMBL;Acc:D6X1U3] |
| TC012437 | 0.80 | 2.42E-02 | 4.27E-17 | NADPH--cytochrome P450 reductase[Source:UniProtKB/TrEMBL;Acc:D6X2B1] |
| TC012506 | 1.30 | 8.51E-04 | 6.38E-05 | Putative uncharacterized protein[Source:UniProtKB/TrEMBL;Acc:D6X2T7] |
| TC012517 | 0.70 | 4.74E-02 | 3.63E-07 | Putative uncharacterized protein[Source:UniProtKB/TrEMBL;Acc:D6X308] |
| TC012529 | 0.84 | 4.05E-02 | 3.63E-07 | Putative uncharacterized protein[Source:UniProtKB/TrEMBL;Acc:D6X327] |
| TC012551 | 0.83 | 3.71E-02 | 7.58E-04 | Putative uncharacterized protein[Source:UniProtKB/TrEMBL;Acc:D6X360] |
| TC012566 | 1.57 | 1.37E-04 | 3.75E-39 | Putative uncharacterized protein[Source:UniProtKB/TrEMBL;Acc:D6X387] |
| TC012691 | 0.84 | 2.17E-02 | 2.06E-04 | Putative uncharacterized protein[Source:UniProtKB/TrEMBL;Acc:D6WZL6] |
| TC013030 | 0.86 | 1.80E-02 | 3.40E-31 | Putative uncharacterized protein[Source:UniProtKB/TrEMBL;Acc:D6WJP0] |
| TC013085 | 0.84 | 2.64E-02 | 3.94E-10 | Putative uncharacterized protein[Source:UniProtKB/TrEMBL;Acc:D6WJ64] |
| TC013230 | 0.79 | 3.35E-02 | 1.78E-04 | Putative uncharacterized protein[Source:UniProtKB/TrEMBL;Acc:D6WMJ1] |
| TC013311 | 0.74 | 4.25E-02 | 1.02E-124 | Putative uncharacterized protein[Source:UniProtKB/TrEMBL;Acc:D6WMA3] |
| TC013486 | 1.26 | 6.39E-04 | 5.68E-04 | Putative uncharacterized protein[Source:UniProtKB/TrEMBL;Acc:D6WL90] |
| TC013560 | 2.15 | 3.73E-03 | 2.52E-10 | Putative uncharacterized protein[Source:UniProtKB/TrEMBL;Acc:D6WKV1] |
| TC013967 | 1.22 | 1.82E-03 | 5.18E-66 | Putative uncharacterized protein[Source:UniProtKB/TrEMBL;Acc:D6WNT5] |
| TC014069 | 0.93 | 1.09E-02 | 2.90E-40 | Putative uncharacterized protein ML4[Source:UniProtKB/TrEMBL;Acc:D6WK07] |
| TC014089 | 1.65 | 1.95E-02 | 4.51E-08 | Putative uncharacterized protein[Source:UniProtKB/TrEMBL;Acc:D6WK73] |
| TC014135 | 0.92 | 3.01E-02 | 1.32E-04 | Putative uncharacterized protein[Source:UniProtKB/TrEMBL;Acc:D6WKF6] |
| TC014177 | 0.83 | 4.00E-02 | 4.91E-04 | Putative uncharacterized protein[Source:UniProtKB/TrEMBL;Acc:D6W6V9] |
| TC014248 | 1.05 | 4.07E-03 | 4.91E-04 | Putative uncharacterized protein[Source:UniProtKB/TrEMBL;Acc:D6WKU9] |
| TC014498 | 1.64 | 1.32E-04 | 2.13E-06 | Putative uncharacterized protein[Source:UniProtKB/TrEMBL;Acc:D6WMB1] |
| TC014499 | 1.86 | 1.06E-04 | 4.65E-91 | Putative uncharacterized protein[Source:UniProtKB/TrEMBL;Acc:D6WMB2] |
| TC014500 | 3.32 | 2.50E-16 | 1.10E-17 | Putative uncharacterized protein[Source:UniProtKB/TrEMBL;Acc:D6WMB3] |
| TC014541 | 0.83 | 2.53E-02 | 3.26E-33 | Putative uncharacterized protein[Source:UniProtKB/TrEMBL;Acc:D6WMC9] |
| TC014548 | 1.28 | 3.49E-03 | 8.06E-06 | Putative uncharacterized protein[Source:UniProtKB/TrEMBL;Acc:D6WME3] |
| TC014742 | 1.61 | 7.51E-03 | 3.93E-29 | Putative uncharacterized protein[Source:UniProtKB/TrEMBL;Acc:D6WJ86] |
| TC014771 | 1.52 | 3.93E-04 | 1.14E-04 | Putative uncharacterized protein[Source:UniProtKB/TrEMBL;Acc:D6WJM8] |
| TC014875 | 1.49 | 4.79E-02 | 4.25E-04 | Putative uncharacterized protein GLEAN_14875[Source:UniProtKB/TrEMBL;Acc:D2A493] |
| TC015222 | 0.95 | 2.63E-02 | 5.98E-06 | Putative uncharacterized protein[Source:UniProtKB/TrEMBL;Acc:D6WWV5] |
| TC015311 | 0.78 | 4.68E-02 | 1.94E-05 | Putative uncharacterized protein GLEAN_15311[Source:UniProtKB/TrEMBL;Acc:D2A4T3] |
| TC015330 | 1.97 | 1.93E-04 | 1.94E-05 | Putative uncharacterized protein GLEAN_15330[Source:UniProtKB/TrEMBL;Acc:D2A4P6] |
| TC015405 | 1.58 | 1.31E-02 | 8.80E-15 | Putative uncharacterized protein GLEAN_15405[Source:UniProtKB/TrEMBL;Acc:D2A4Q6] |
| TC015433 | 1.27 | 6.36E-03 | 1.47E-13 | Putative uncharacterized protein GLEAN_15433[Source:UniProtKB/TrEMBL;Acc:D2A4W4] |
| TC015479 | 0.73 | 4.01E-02 | 3.33E-14 | Putative uncharacterized protein GLEAN_15479[Source:UniProtKB/TrEMBL;Acc:D2A563] |
| TC015663 | 0.87 | 1.98E-02 | 1.27E-13 | Putative uncharacterized protein GLEAN_15663[Source:UniProtKB/TrEMBL;Acc:D2A693] |
| TC015689 | 0.96 | 1.29E-02 | 7.32E-05 | Putative uncharacterized protein GLEAN_15689[Source:UniProtKB/TrEMBL;Acc:D2A6F2] |
| TC015719 | 3.36 | 2.05E-14 | 7.01E-10 | Putative uncharacterized protein GLEAN_15719[Source:UniProtKB/TrEMBL;Acc:D2A3N2] |
| TC015725 | 1.32 | 1.04E-02 | 6.35E-66 | Putative uncharacterized protein GLEAN_15725[Source:UniProtKB/TrEMBL;Acc:D2A3N8] |
| TC015749 | 1.87 | 1.13E-04 | 1.45E-05 | Putative uncharacterized protein GLEAN_15749[Source:UniProtKB/TrEMBL;Acc:D2A3S7] |
| TC015750 | 2.06 | 3.46E-07 | 2.36E-04 | Putative uncharacterized protein GLEAN_15750[Source:UniProtKB/TrEMBL;Acc:D2A3S9] |
| TC015751 | 2.03 | 2.64E-06 | 2.36E-04 | Putative uncharacterized protein GLEAN_15751[Source:UniProtKB/TrEMBL;Acc:D2A3T0] |
| TC015822 | 1.57 | 4.70E-04 | 9.99E-04 | Putative uncharacterized protein GLEAN_15822[Source:UniProtKB/TrEMBL;Acc:D2A446] |
| TC015854 | 0.98 | 1.75E-02 | 1.45E-74 | Putative uncharacterized protein GLEAN_15854[Source:UniProtKB/TrEMBL;Acc:D2A4B0] |
| TC016160 | 0.93 | 4.82E-02 | 0.00E+00 | Putative uncharacterized protein[Source:UniProtKB/TrEMBL;Acc:D6WBH4] |
| TC016203 | 0.77 | 3.06E-02 | 1.07E-05 | Amidophosphoribosyltransferase[Source:UniProtKB/TrEMBL;Acc:D6X4R0] |
| TC016225 | 0.80 | 2.38E-02 | 4.68E-05 | Putative uncharacterized protein[Source:UniProtKB/TrEMBL;Acc:D6X4R1] |
| TC016246 | 2.62 | 1.52E-02 | 2.04E-04 | Putative uncharacterized protein[Source:UniProtKB/TrEMBL;Acc:D7ELN5] |
| TC016332 | 1.00 | 7.66E-03 | 4.80E-11 | Putative uncharacterized protein[Source:UniProtKB/TrEMBL;Acc:D7GYM9] |
| TC016380 | 0.88 | 2.65E-02 | 0.00E+00 | Putative uncharacterized protein[Source:UniProtKB/TrEMBL;Acc:D6WPD4] |
| TC016388 | 0.83 | 4.52E-02 | 8.06E-12 | Putative uncharacterized protein[Source:UniProtKB/TrEMBL;Acc:D7ELV5] |
| TC030482 | 1.85 | 1.15E-03 | 7.48E-04 | Antimicrobial peptide[Source:UniProtKB/TrEMBL;Acc:D6W9Z0] |
| TC030588 | 0.78 | 3.15E-02 | 3.54E-07 | Putative uncharacterized protein GLEAN_14981-OG11719 [Source:UniProtKB/TrEMBL;Acc:D2A6I0] |
